# *Bifidobacterium*-derived short-chain fatty acids and indole compounds attenuate nonalcoholic fatty liver disease by modulating gut-liver axis

**DOI:** 10.3389/fmicb.2023.1129904

**Published:** 2023-03-01

**Authors:** Sang Jun Yoon, Jeong Seok Yu, Byeong Hyun Min, Haripriya Gupta, Sung-Min Won, Hee Jin Park, Sang Hak Han, Byung-Yong Kim, Kyung Hwan Kim, Byoung Kook Kim, Hyun Chae Joung, Tae-Sik Park, Young Lim Ham, Do Yup Lee, Ki Tae Suk

**Affiliations:** ^1^Institute for Liver and Digestive Disease, Hallym University, Chuncheon, Republic of Korea; ^2^Department of Agricultural Biotechnology, Center for Food and Bioconvergence, Research Institute of Agricultural and Life Sciences, Seoul National University, Seoul, Republic of Korea; ^3^Department of Pathology, Hallym University College of Medicine, Chuncheon, Republic of Korea; ^4^Chong Kun Dang Healthcare Institute, Seoul, Republic of Korea; ^5^Chong Kun Dang Bio Research Institute, Gyeonggi-do, Republic of Korea; ^6^Department of Life Science, Gachon University, Sungnam, Republic of Korea; ^7^Department of Nursing, Daewon University College, Jecheon-si, Republic of Korea

**Keywords:** nonalcoholic fatty liver disease, gut microbiota, probiotics, metagenomics, gut-liver axis

## Abstract

Emerging evidences about gut-microbial modulation have been accumulated in the treatment of nonalcoholic fatty liver disease (NAFLD). We evaluated the effect of *Bifidobacterium breve* and *Bifidobacterium longum* on the NAFLD pathology and explore the molecular mechanisms based on multi-omics approaches. Human stool analysis [healthy subjects (*n* = 25) and NAFLD patients (*n* = 32)] was performed to select NAFLD-associated microbiota. Six-week-old male C57BL/6 J mice were fed a normal chow diet (NC), Western diet (WD), and WD with *B. breve* (BB) or *B. longum* (BL; 109 CFU/g) for 8 weeks. Liver/body weight ratio, histopathology, serum/tool analysis, 16S rRNA-sequencing, and metabolites were examined and compared. The BB and BL groups showed improved liver histology and function based on liver/body ratios (WD 7.07 ± 0.75, BB 5.27 ± 0.47, and BL 4.86 ± 0.57) and NAFLD activity scores (WD 5.00 ± 0.10, BB 1.89 ± 1.45, and BL 1.90 ± 0.99; *p* < 0.05). Strain treatment showed ameliorative effects on gut barrier function. Metagenomic analysis showed treatment-specific changes in taxonomic composition. The community was mainly characterized by the significantly higher composition of the *Bacteroidetes* phylum among the NC and probiotic-feeding groups. Similarly, the gut metabolome was modulated by probiotics treatment. In particular, short-chain fatty acids and tryptophan metabolites were reverted to normal levels by probiotics, whereas bile acids were partially normalized to those of the NC group. The analysis of gene expression related to lipid and glucose metabolism as well as the immune response indicated the coordinative regulation of β-oxidation, lipogenesis, and systemic inflammation by probiotic treatment. BB and BL attenuate NAFLD by improving microbiome-associated factors of the gut-liver axis.

## Introduction

Nonalcoholic fatty liver disease (NAFLD) is a leading cause of chronic liver disease and one of the major public health problems (Younossi, 2019). The pathology is mostly prevalent not only in obese and diabetic patients but also in nondiabetic and lean individuals (Younossi et al., 2012). The progression of NAFLD is triggered by an increased synthesis and reduced utilization of lipids, which results in excessive deposition of triglyceride-rich lipid droplets in the liver, commonly termed hepatic steatosis (Cohen et al., 2011). Metabolic dysregulation leads to cellular stresses, such as oxidative stress, and consequently induces hepatic inflammation that can progress into nonalcoholic steatohepatitis (NASH; Kleiner and Makhlouf, 2016) and further into severe forms of liver diseases, including cirrhosis and hepatocellular carcinoma (Debes et al., 2020).

Dietary patterns high in fat are the most important risk factors for the development and progression of NAFLD (Yang et al., 2020). Therefore, the diet-induced animal model for NAFLD is being widely applied. A western diet (WD), which is high in saturated fat, has been repeatedly demonstrated to be efficient in designing preclinical models for NAFLD due to its relative simplicity and ability to trigger pathological outcomes similar to those in humans (Yang et al., 2020). While the underlying mechanisms are largely unknown, recent studies have reported that high-fat diet-related liver injuries are accompanied by higher expression of lipogenesis genes, reduced expression of β-oxidation genes, elevated production of pro-inflammatory cytokines and reactive oxygen species, and alteration of the gut microbiome (Leung et al., 2016).

The impact of the WD on the complex interactions between gut microbes and the host has become an interesting area of medical research. There is a general understanding that the WD greatly influences the composition and function of gut microbiota. WD increase *Firmicutes*/*Bacteroidetes* ratio and this change is driven by increases in *Erysipelotrichales*, *Bacilli*, and *Clostridiales* (*Firmicutes*; Malesza et al., 2021). However, little is known about the pathological mechanisms and roles of WD-induced gut dysbiosis during the progression of NAFLD. The basic idea lies in the roles of microbial antigens, production of microbe-derived metabolites, and intestinal permeability along with translocation to the portal vein. As the liver obtains most of its blood flow from the intestine, it is highly exposed to these microbial products as well as the intact microbes themselves. Therefore, the gut microbiome-liver axis can be used as a target for therapeutic interruptions of NAFLD.

Probiotics are believed to delay the progression of NAFLD with therapeutic endpoints, such as modulation of gut microbiota, intestinal permeability, and inflammatory pathways. This is a lucrative choice considering its simple availability, cost convenience, and absence of severe side effects. Different *Bifidobacterium* species have been proven effective treatments for hepatic steatosis and inflammation, acute liver injury and cirrhosis (Yan et al., 2020). *Bifidobacterium breve* (BB) and *B. longum* (BL) are some of the commonly used probiotic species. They are generally dominant in infants and were first isolated from the feces of breast-fed newborns (Milani et al., 2017). Both species have been reported to possess an array of enzymes that enable them to adapt and compete in an environment with changing nutritional conditions, such as the gut. A strain from BB has been recently reported to suppress body weight gain and fat deposition in a dose-dependent manner accompanied by a reduced level of serum total cholesterol (Minami et al., 2018). Similarly, BL has also been shown to attenuate liver fat accumulation, lower serum total cholesterol, and induce growth of *Bacteroides* in rats fed a high-fat diet (Yin et al., 2010). However, the mechanisms by which *Bifidobacterium* strains exert their attenuating effects on NAFLD, especially regarding modulation of the gut microbiota, are poorly understood.

This study aimed to elucidate the WD-gut microbiome-liver axis along with an evaluation of probiotic interruptions. This was conducted by establishing a mouse NAFLD model using a WD challenge, investigating the effects on gut microbiota, examining the underlying mechanisms by which alteration of gut microbiota is involved in progression of NAFLD, and evaluating the effects of the probiotics BB and BL with a focus on gut modulation and reduction of lipogenesis and inflammatory responses.

## Results

### Distinct gut-microbial compositions in NAFLD patients

Altogether, a total of 57 human subjects (healthy controls, *n* = 25 and NAFLD patients, *n* = 32) were included in this study. Differences in gut-microbial compositions and functional biomarkers were compared between the individuals with and without NAFLD. The microbiota compositions and relative abundances of functional markers were significantly altered in the NAFLD group compared to those of healthy control subjects.

**Figure 1:**
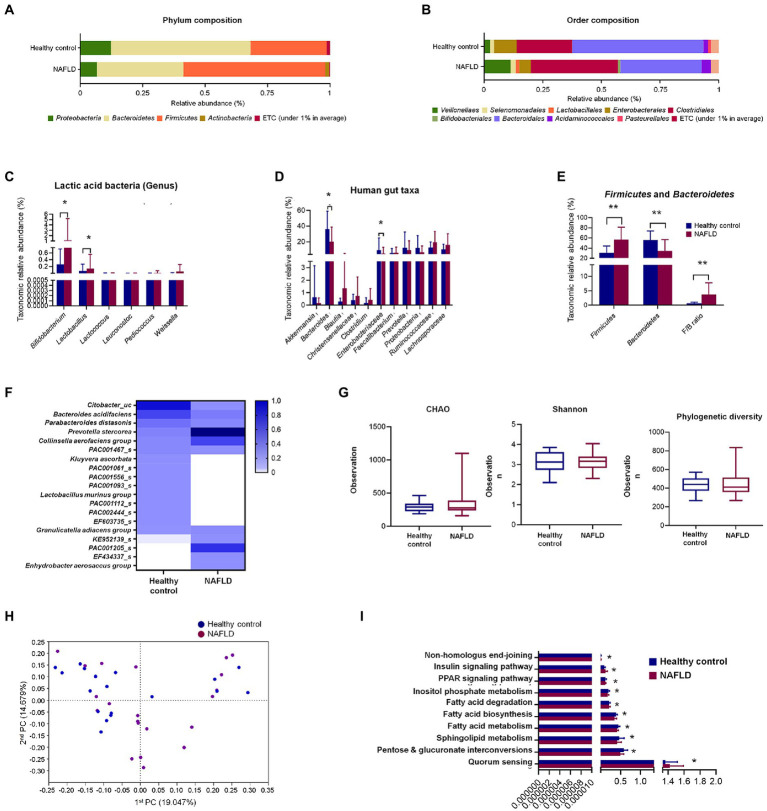
Comparisons of relative abundances of fecal microbiota and functional biomarkers between healthy control and NAFLD patients. **(A)** Structural comparisons of microbial compositions at phylum level. **(B)** Structural comparisons of microbial compositions at order level. **(C)** Taxonomic relative abundances of individual lactic acid bacteria. **(D)** Taxonomic relative abundances of individual phyla. **(E)** Taxonomic relative abundances and ratio of *Firmicutes* and *Bacteroidetes*. **(F)** Heat map showing taxonomic relative abundances of individual species. **(G)** Alpha diversity based on CHAO and Shannon indexes and phylogenetic diversity. **(H)** PCA plot representing beta diversity. **(I)** Functional biomarker analysis. NAFLD, Nonalcoholic fatty liver disease; PCA, principal component analysis. Independent *t*-test: *p < 0.05 and **p < 0.01.

At the phylum level, *Bacteroidetes* (56%) were predominant in the healthy control subjects, followed by *Firmicutes* (31%), while *Firmicutes* (59%) were dominant, followed by *Bacteroidetes* (35%) in the NAFLD patients (Figure 1A). Similarly, the composition at the order level showed noticeable changes in NAFLD, with *Bacteroidales* being predominant in healthy controls and *Clostridiales* and *Bacteroidales* having similar abundances in the case of NAFLD (Figure 1B).

At the genus level, six lactic acid bacteria were separately compared between healthy controls and NAFLD patients. *Bifidobacterium* and *Lactobacillus* showed significant variation between healthy controls and NAFLD patients, while no significant difference was observed for *Lactococcus*, *Leuconostoc*, *Pediococcus,* and *Weissella* genera (Figure 1C). Additionally, we separately compared the relative abundances of 11 more individual genera and family, namely, *Akkermansia*, *Bacteroides*, *Blautia*, *Christensenellaceae*, *Clostridium*, *Enterobacteriaceae*, *Faecalibacterium*, *Prevotella*, *Proteobacteria*, *Ruminococcaceae,* and *Lachnospiraceae,* among which only *Bacteroides* and *Enterobacteriaceae* showed significant reduction in NAFLD patients (Figure 1D). In *Firmicutes*-to-*Bacteroidetes* ratio (F/B ratio) result, *Firmicutes* level, *Bacteroidetes* level, and F/B ratio shows the significant difference between the healthy control group and NAFLD patients’ group (Figure 1E). However, no significant differences were observed in alpha diversities based on CHAO and Shannon indices and phylogenetic diversity between the two groups (Figure 1G). Clear differences were observed in the heat map profiles of the relative abundances of most genera, and noticeable discriminations were visible between the healthy and NAFLD groups during principal component analysis (PCA; Figures 1F,H). No significant differences between the two groups were observed during biomarker analysis of 10 pathways (Figure 1I).

### Supplementation with *Bifidobacterium* strains ameliorates the progression of NAFLD

**Figure 2:**
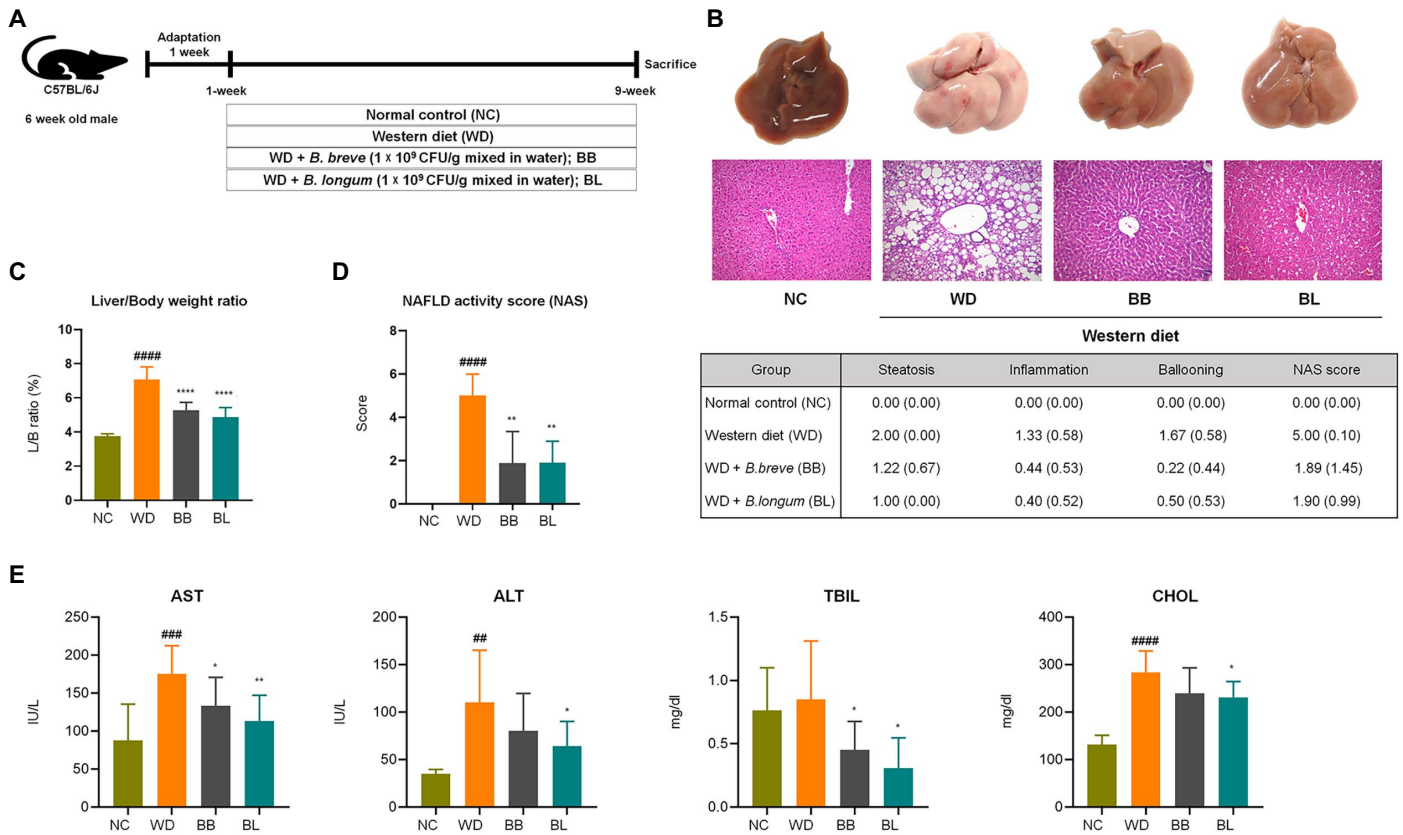
*Bifidobacterium breve* and *Bifidobacterium longum* supplement on western diet-induced NAFLD in mice. **(A)** Experiment design depicting the animal model used. **(B)** Top: Representative liver specimens of gross examinations; Bottom: representative H&E-stained liver sections. **(C)** Liver weight to body weight ratio. **(D)** NAS. Left: Bar graph representation; Right: Individual gradings showing steatosis, inflammation, and ballooning score. H&E-stained liver sections were assessed blindly by an experienced liver pathologist for steatosis, hepatocyte ballooning and lobular inflammation. **(E)** Serum levels of liver function test enzymes and cholesterol. NAFLD, nonalcoholic fatty liver disease; NC, normal chow diet group; WD, western diet group; BB, WD + *B. breve* group; BL, WD + *B. longum* group; NAS, NAFLD activity score; AST, aspartate aminotransferase; ALT, alanine aminotransferase; TBIL, total bilirubin; CHOL, total cholesterol. # obtained statistics by comparing ND and WD. * statistics were obtained by comparing WD with the experimental group. One-way analysis of variance (ANOVA): ^##^*p* < 0.01, ^###^*p* < 0.001, ^####^*p* < 0.0001, ^*^p < 0.05, ^**^p < 0.01, and ^****^p < 0.0001.

The animal experimental design is described in Figure 2A. Mice were fed a normal chow (NC) diet, WD (42% fat), or WD supplemented with probiotic strains, BB and BL for 9 weeks. Increased rates of hepatic lipogenesis and resultant steatosis were determined in the WD group by histological evaluations of the accumulation of triglycerides in hepatocytes (Adams and Angulo, 2005). As shown in the gross liver images and H&E staining results, the livers of the WD group animals showed a noticeable increase in size along with a strikingly whitish appearance caused by the accumulation of lipid droplets, indicating drastic liver steatosis, while the livers of mice fed a normal diet remained dark and of normal size (Figure 2B). Similarly, histological assessments using H&E staining showed severe vacuolations of liver tissue in the WD group characterized by both microvascular and macrovascular steatosis because of lipid deposition in hepatocytes.

The average body weight of the WD group was significantly higher (*p* < 0.05) than that of the NC group, which corresponded with the substantial increase in liver weight and liver-to-body weight (L/B) ratio (*p* < 0.05) compared to the NC group (Figure 2C). Quantitative evaluations of steatosis stage and necroinflammation activity were estimated from H&E staining of liver sections based on standard histological scoring methods. The steatosis score, hepatitis score, and NAFLD activity score (NAS) were significantly higher (*p* < 0.05) in the WD group than in the NC group (Figure 2D). We further analyzed serum levels of aspartate transaminase (AST), alanine transaminase (ALT), total bilirubin (TBIL), and total cholesterol (CHOL) to evaluate liver function. Significant increases in AST, ALT, and CHOL levels were observed in the WD group compared to those in the NC group (Figure 2E). Probiotic supplementation with *B. breve* and *B. longum* significantly ameliorated the progression of hepatic steatosis. Both strains resulted in a significant reduction in the L/B ratio and a close to complete remission of steatosis. Similarly, both probiotic strains resulted in substantial reductions in AST and TBIL levels compared to the WD group. Both probiotic strains were able to reduce the gain of fat mass and hepatic lipid accumulation, which also showed a positive correlation with liver enzyme analysis.

### Gut-microbial community varies by different treatments

**Figure 3:**
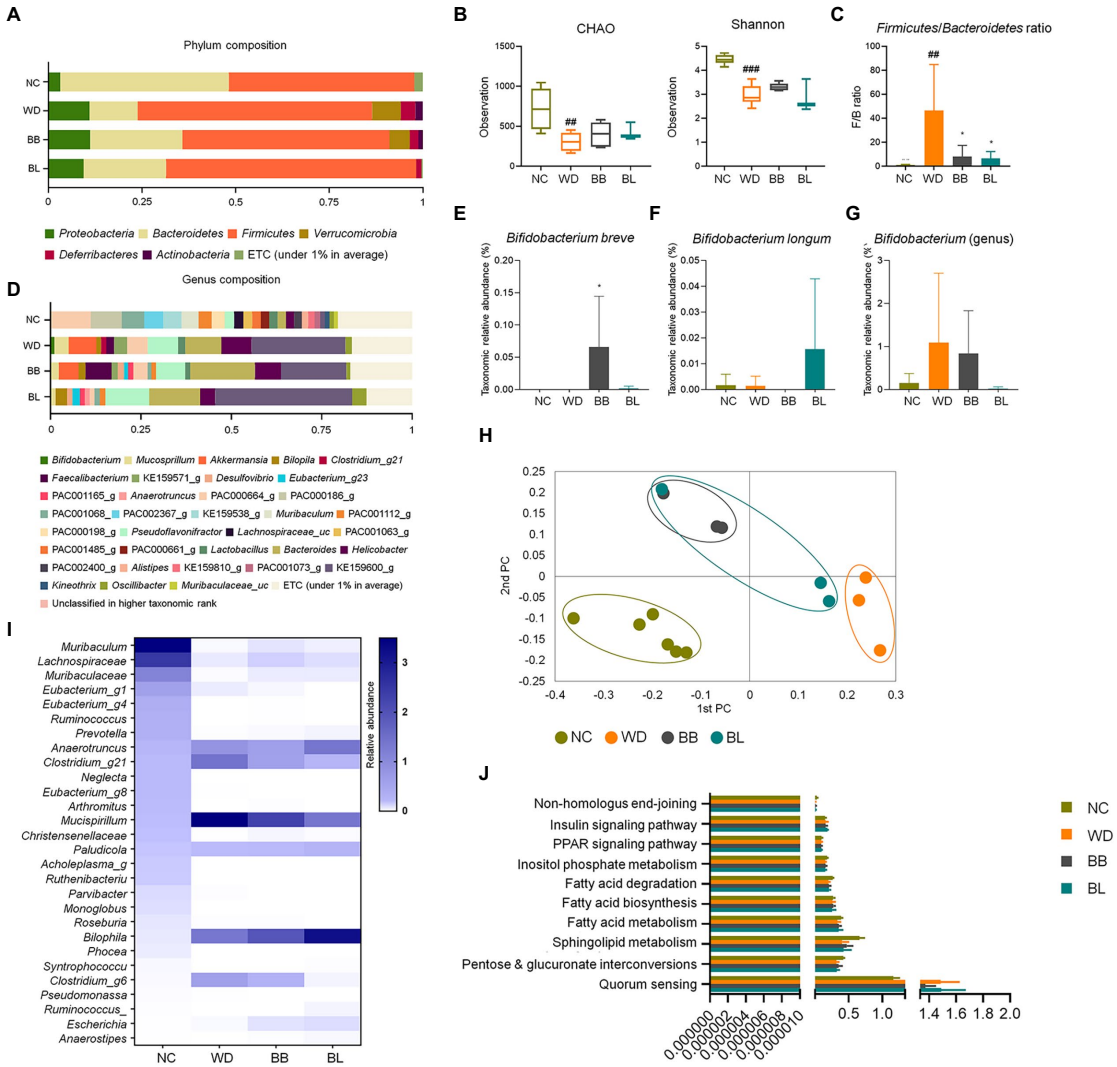
Comparisons of relative abundances of fecal microbiota and functional biomarkers between normal diet, western diet model, and probiotic supplementation in mice NAFLD model. **(A)** Structural comparisons of microbial compositions at phylum level. **(B)** Alpha diversity based on CHAO and Shannon indexes. **(C)** Ratio of taxonomic relative abundances *Firmicutes* and *Bacteroidetes*. **(D)** Structural comparisons of microbial compositions at genus level. **(E)** *Bifidobacterium breve* species level competition. **(F)** *Bifidobacterium longum* species level competition. **(G)** *Bifidobacterium* genus level competition. **(H)** PCA plot representing beta diversity. **(I)** Heat map showing taxonomic relative abundances of individual species. **(J)** Functional biomarkers. NAFLD, nonalcoholic fatty liver disease; NC, normal chow diet group; WD, western diet group; BB, WD + *B. breve* group; BL, WD + *B. longum* group. # obtained statistics by comparing ND and WD. * statistics were obtained by comparing WD with the experimental group. One-way analysis of variance (ANOVA): ^##^*p* < 0.01, ^###^*p* < 0.001, and ^*^p < 0.05.

The compositions at the phylum level differed significantly among the four groups (NC, WD, BB, and BL; Figure 3A). *Firmicutes* (63%) was significantly enriched in the WD and BL (67%) groups compared to the NC (50%) and BB (55%) groups, while *Bacteroidetes* (45%) composition was more abundant in the NC group than in compared to all the other groups. The WD group also featured a higher composition of *Proteobacteria*, *Verrucomicrobia*, *Deferribacteres*, and *Actinobacteria* than the NC group. The BB and BL groups presented transitional patterns of the phyla composition between the NC and WD groups.

The F/B ratios of the probiotic-feeding groups were marginally lower than that of the WD group, where the *Firmicutes* composition was similar, but the *Bacteroidetes* composition was more enriched. The profiles of 16S rRNA gene amplicon sequencing were comparably analyzed to investigate characteristic changes in the gut-microbial composition. In the alpha diversity analysis based on CHAO and Shannon indices, the WD, BB, and BL groups had reduced microbial richness relative to the NC group, but no significant difference was observed between the WD group and probiotic-supplemented groups (Figure 3B). During a separate comparison of the F/B ratio between groups, the ratio in the WD group (4.9) was substantially higher than that in the NC group (1.1; Figure 3C). A remarkable reduction in the F/B ratio was obtained in the probiotic supplementation groups. The bacterial composition at the genus level also showed a significant difference between the control group and WD, with slightly distinct profiles observed between the WD and treatment groups (Figure 3D).

The most pathologically relevant observation was a noticeable increase in *Helicobacter* in the WD group. *Helicobacter* is a member of the phylum *Proteobacteria,* and an increase in the relative abundances of members of this genus has been reported to alter immune homeostasis in mice (Ray et al., 2015). The relative abundance of this genus increased from 2% in the NC group to 8.3% in the WD group, and a slight reduction was seen in both treatment groups, with BL showing a more significant reduction. Similar patterns were observed for the genus *Pseudoflavonifractor,* where its relative abundance markedly increased in the WD and probiotic groups. While a remarkable increase in *Bacteroides* was observed in the WD group, supplementation with both strains showed no effect. Separate analysis of the relative abundance of the individual probiotic strains used also showed results that confirmed the validity of the effect of the supplementation, where each strain was predominant in the respective groups (Figures 3E–G).

The beta-diversity analysis using PCoA based on the Bray–Curtis dissimilarity matrix demonstrated clear discrimination of the NC group from the other groups (Figure 3H). Heat map analysis of 28 identified genera showed clear discrimination for NC vs. WD and both treatment groups. Probiotic treatments were able to result in slight modulations for a few bacterial members (Figure 3I). Biomarker analysis was performed for the same pathways as in the human samples. Similar to the human fecal analysis, there was no significant difference between the four mouse groups (Figure 3J).

### The gut metabolome is substantially normalized by strain treatment

**Figure 4:**
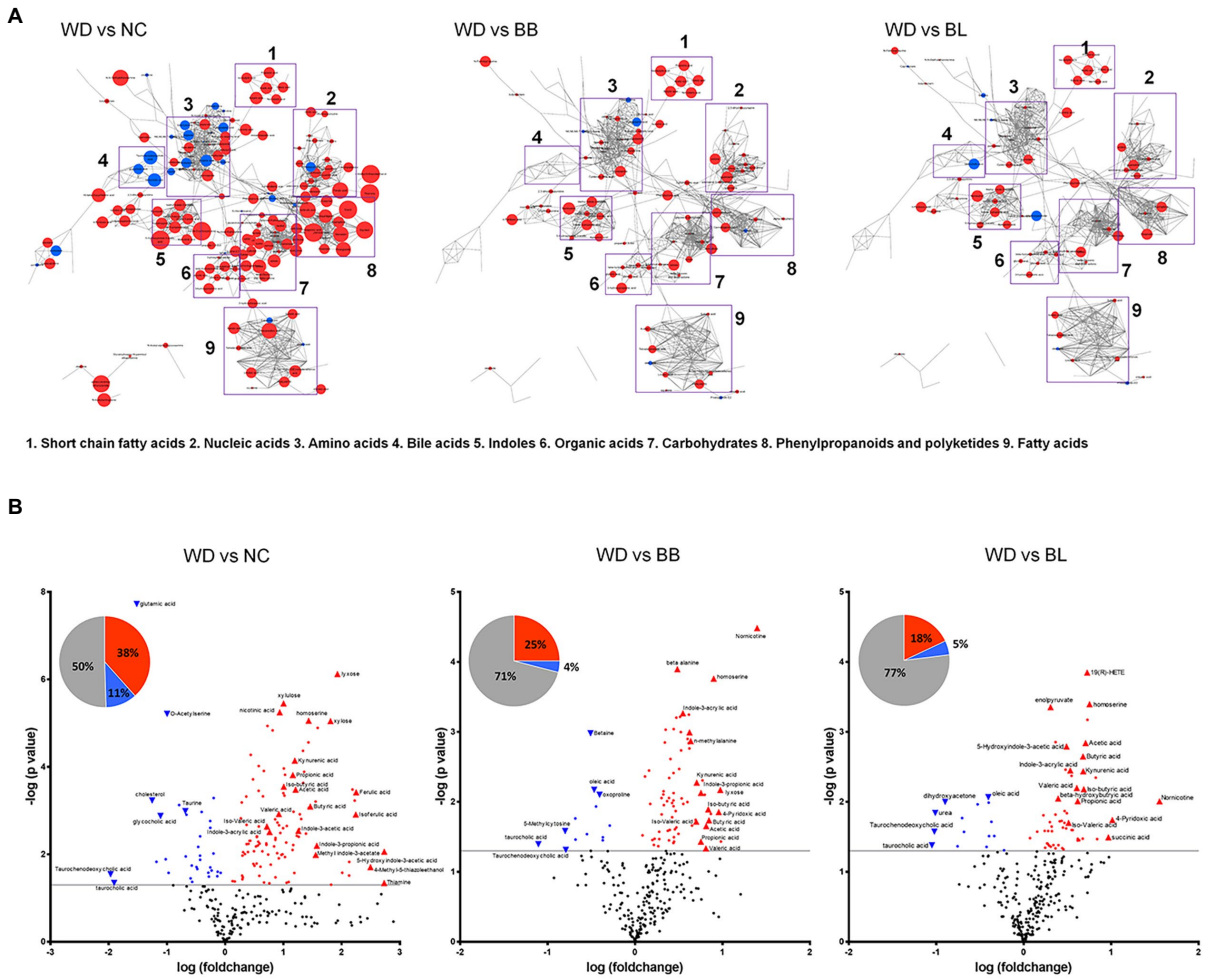
Characteristic alteration of gut metabolomic profiles by *Bifidobacterium* supplementation. **(A)** Overview of the metabolic features. The network is constructed based on chemical structural similarity (Tanimoto score) and KEGG reaction pair (substrate-product relation), which results in distinctive metabolic modules indicated by box. Red and blue colors present significantly higher or lower abundant in NC, BB, and BL groups, respectively, compared to WD (Student’s *t*-test, *p* < 0.05). Node sizes are determined by the ratios. **(B)** Pie charts present the number of metabolites that were significantly different in other groups, respectively, compared to WD (Student’s *t*-test, *p* < 0.05). Red and blue colors present significantly higher or lower abundance in other groups, respectively, compared to WD (*p* < 0.05). Volcano plot for identification of metabolites with significant differences in the NC, BB, and BL, respectively, compared to WD group. The X-axis presents the fold change in the log_10_ scale, and the Y-axis indicates the statistical significance (value of *p*) in the log_10_ scale based on Student’s *t*-test. NC, normal chow diet group; WD, western diet group; BB, WD + *Bifidobacterium breve* group; BL, WD + *Bifidobacterium longum* group.

Targeted and untargeted metabolomics was applied to acquire the comprehensive profiles of the cecal metabolites. The metabolic profiles were collected from the NC, WD, BB, and BL groups. The metabolic signals were structurally identified, which resulted in 290 unique primary and secondary metabolites. The identified metabolites were categorized by chemical ontology analysis as follows: organic acids (26%), lipids (19%), organic oxygen compounds (16%), and organoheterocyclic compounds (14%) at the superclass level (Figure 4A; Supplementary Figure 2A).

The metabolic profiles of the four groups were characterized based on principal component analysis (PCA). Similar to the microbial taxonomic profiles, a clear discrimination was determined between NC and the other groups (Supplementary Figure 2B). To provide an overview of the characteristic metabolic classes according to the different treatments, chemical enrichment analysis was conducted, which provided comprehensive classification with statistical criteria based on chemical similarity and ontology mapping (Lee B. M. et al., 2020). The map consisted of 15 major clusters as follows: hexoses, pyridines, fatty acids, sugar alcohols, pyrimidinones, azoles, disaccharides, sugar acids, hydroxybutyrates, dicarboxylic acids, indoles, butyrate, pyrimidine nucleosides, amino acids, and cholestenes. The enrichment analysis demonstrated chemical class-wise quantitative features of the NC, BB, and BL groups compared to the WD group (Supplementary Figures 1, 2C). Compared to the WD groups, the NC and probiotic-feeding groups showed increases in the metabolic modules of amino acids, indoles, and butyrates. A decrease in the taurine-conjugation class was a common feature for the NC, BB, and BL groups. The NC group showed specific changes in the modules of hexose, sugar alcohol, disaccharides, pyrimidine nucleosides, basic amino acids, cholestenes, and azoles, whereas an alteration in the module of unsaturated fatty acids was specific to the BB group.

We further verified the metabolites that were significantly different in the NC, BB, and BL groups compared to those in the WD group. Among the 290 metabolites, 147 metabolites were significantly different in the NC group compared to those in the WD group. Approximately 38% of metabolites were significantly higher in the NC group than in the WD group, and 5-hydroxyindole-3-acetic acid, thiamine, and 4-methyl-5-thiazole ethanol showed the largest differences. In contrast, taurochenodeoxycholic acid and taurocholic acid presented the highest upregulation in the WD group (Figure 4B). The BB and BL groups showed significantly higher levels in 25 and 18% of metabolites, respectively, while 11% and 4% of metabolites were at substantially lower abundance, respectively, compared to those in the WD group (Figure 4B). Note that SCFAs (butyric acid and acetic acid), indole compounds (indone-3-propionic acid and methyl indole-3-phosphate), and bile acids (taurodeoxycholic acid and taurocholic acid) were associated with the level of the NC group with the highest fold-changes compared to that of the WD group. Pairwise metabolomic comparison between NC group and probiotic-feeding groups (BB and BL) provided in Supplementary Table 1.

### Gut microbiota-derived metabolites are partially normalized by *Bifidobacterium* supplementation

**Figure 5:**
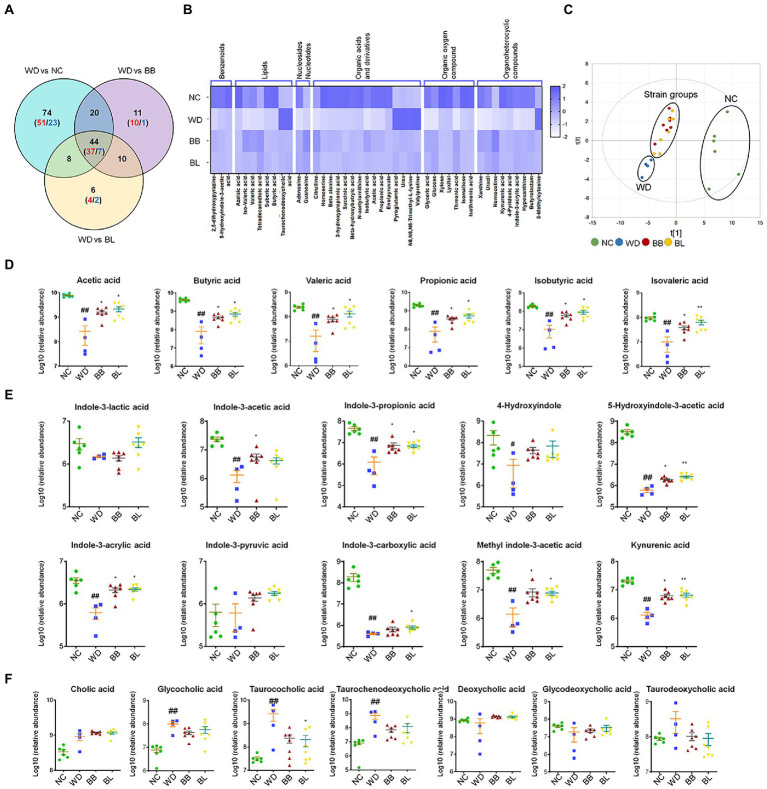
Gut microbiota-derived common metabolic signatures. **(A)** Venn diagram of common and unique metabolites among the NC, BB, and BL groups as compared to the WD group. Statistical significance is determined based on Student’s *t*-test (*p* < 0.05). **(B)** Heat map showing common metabolites that are classified into 7 superclasses. **(C)** core scatter plot of the common metabolites by PCA. **(D)** Relative abundance of cecal SCFAs. **(E)** Relative abundance of cecal tryptophan metabolites. **(F)** Relative abundance of cecal bile acids. Statistical significance is determined based on Mann–Whitney *U*-test. NC, normal chow diet group; WD, western diet group; BB, WD + *Bifidobacterium breve* group; BL, WD + *Bifidobacterium longum* group; PCA, principal component analysis; SCFA, short-chain fatty acid. # obtained statistics by comparing ND and WD. * statistics were obtained by comparing WD with the experimental group. One-way analysis of variance (ANOVA): ^#^*p* < 0.05, ^##^*p* < 0.01, ^*^p < 0.05, and ^**^p < 0.01.

Next, we investigated common metabolic signatures assuming that the metabolites similarly regulated between the NC and probiotic-feeding groups may play key roles in preventing the progression of NAFLD. A total of 44 metabolites showed significant changes that were common among the NC and probiotic-feeding groups (BB and BL) compared to the WD group (*p* < 0.05; Figure 5A). Most of the common metabolites were more enriched in the NC, BB, and BL groups than in the WD group (37 out of 44 common metabolites). The heat map analysis indicated the partial normalization of the common metabolome by the probiotic treatment (Figure 5B). The PCA plot with the score scatterplots of common 33 metabolites of cecal contents showed different discrimination among the NC, WD, and two probiotic-feeding groups (Figure 5C).

Among the common metabolites, short-chain fatty acids (SCFAs), tryptophan metabolites, and bile acids are gut microflora-derived compounds that are directly related to various types of pathology. Accordingly, we analyzed the profiles and the statistical significance across all groups based on the Mann–Whitney *U*-test with adjustment for multigroup comparisons (*p* < 0.05). Indeed, all SCFAs were at significantly higher levels in the NC, BB, and BL groups than in the WD group (Figure 5D). Most tryptophan metabolites showed similar patterns to SCFAs, where the abundances were substantially higher in the NC, BB, and BL groups than in the WD group. The metabolites included indole-3-propionic acid, indole-3-acrlyic acid, 5-hydroxyindole-3-acetic acid, methyl indole-3-acetic acid, and kynurenic acid (Figure 5E). In contrast, marginal differences were determined in bile acids among the four groups. Glycocholic acid, taurocholic acid, and taurochenodeoxycholic acid showed significant differences in the NC group compared to those in the WD group (Figure 5F). Bile acids were found in a similar pattern but showed unsubstantial differences in the BB and BL groups.

### *Bifidobacterium* modulates gene expression associated with lipid metabolism, inflammation, glucose metabolism, immune cell infiltration, and gut barrier function

**Figure 6:**
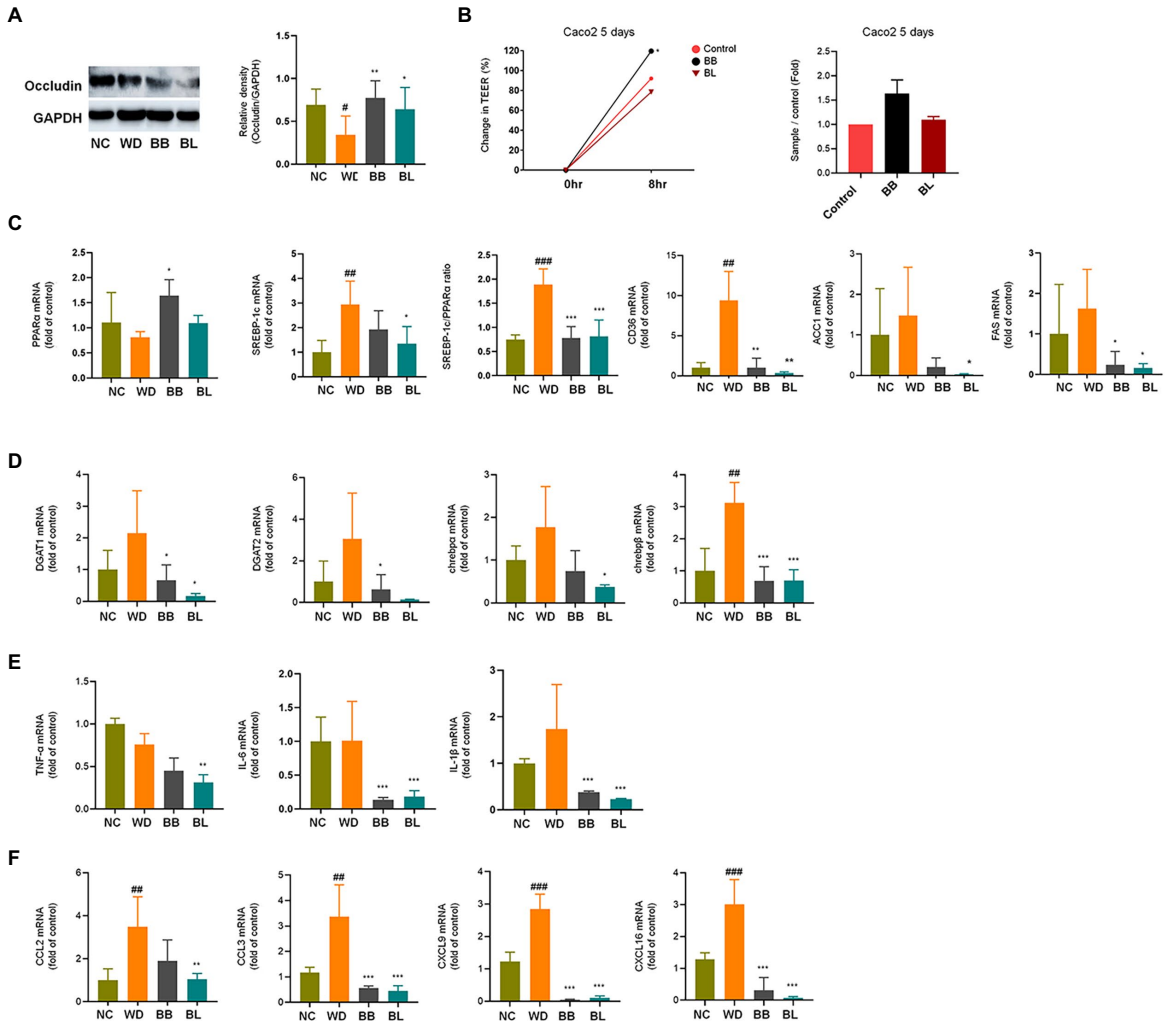
Effects of *Bifidobacterium breve* and *Bifidobacterium longum* supplementation on hepatic lipid metabolism and inflammation, and gut-liver axis markers. **(A)** Western blot analyses of the tight junction protein occludin and GADPH in mice intestine. Left: Representative blots shown with densitometry, right: Quantified results. **(B)** TEER measurements on Caco-2 cells monolayer. Left: Change in TEER, right: Quantified comparison between control and treatment groups. **(C)** mRNA levels of lipid metabolism genes. **(D)** mRNA levels of glucose metabolism genes. **(E)** mRNA levels of pro-inflammatory cytokines. **(F)** mRNA levels of immune cell recruitment chemokines. NC, normal chow diet group; WD, western diet group; BB, WD + *B. breve* group; BL, WD + *B. longum* group; TEER, trans-epithelial electrical resistance; GAPDH, glyceraldehyde 3-phosphate dehydrogenase; PPAR, peroxisome proliferator-activated receptor; SREBP, sterol regulatory element-binding protein; CD, cluster of differentiation; ACC1, acetyl-CoA carboxylase; FAS, fatty acid synthase; DGAT, diglyceride acyltransferase; ChREBP, carbohydrate response element binding protein; TNF, tumor necrosis factor; IL, interleukin; CCL, C-C motif chemokine ligand; CXCL, C-X-C motif chemokine ligand. # obtained statistics by comparing ND and WD. * statistics were obtained by comparing WD with the experimental group. One-way analysis of variance (ANOVA): ^#^*p* < 0.05, ^##^*p* < 0.01, ^###^*p* < 0.001, ^*^p < 0.05, ^**^p < 0.01, and ^***^p < 0.001.

To evaluate the effect of the probiotics *B. breve* and *B. longum* on important NAFLD progression both *in vivo* and *in vitro*, analyses of common biomarkers for hepatic lipid metabolism, inflammation, and gut barrier function were conducted. Western blotting analysis was performed to determine the relative occludin expression in mouse intestine tissue. The results showed that the western diet reduced the expression of occludin in the intestine (Figure 6A). Supplementation with *B. breve* and *B. longum* resulted in a significant increase in its expression, indicating a modulating effect of the strains on gut barrier function. This evidence was strengthened by an increased Trans-epithelial electrical resistance (TEER) measurement during incubation of the probiotic strains on the Caco-2 cell monolayer, which correlated with the western blotting results (Figure 6B). During the determination of hepatic mRNA levels of the selected markers, the western diet significantly upregulated genes related to lipid metabolism (Figure 6C) and glucose metabolism (Figure 6D).

The progression of NAFLD is tightly linked to lipid and glucose metabolism (Gastaldelli et al., 2009). Sterol receptor element-binding protein-1c (SREBP-1c) induces lipogenesis in the liver, while peroxisome proliferator-activated receptor-alpha (PPARα) mediates fatty acid (FA) β-oxidation (Goto et al., 2013). The SREBP-1c/PPARα ratio has been reported as a good marker for hepatic steatosis (Pettinelli et al., 2009). Accordingly, the mRNA levels of these genes in the liver were determined based on qPCR. The WD group showed significantly higher (*p* < 0.001) mRNA levels of SREBP-1c, while the PPARα level was slightly lower than that in the NC group. Consequently, the ratio of SREBP-1c/PPARα was significantly higher (*p* < 0.01) in the WD group than in the NC group. The BB group was characterized by significant (*p* < 0.05) upregulation of PPARα, whereas the BL group showed significant (*p* < 0.001) downregulation of SREBP-1c, which resulted in reduced ratios compared to that of the WD. Similarly, cluster of differentiation (CD) 36 was significantly upregulated in the WD, and both probiotic strains were able to reverse it. No significant difference between the NC and WD groups was observed in the case of acetyl-coenzyme A carboxylase 1 (ACC-1) and fatty acid synthase (FAS), which are also genes that play important roles in lipid metabolism. The mRNA levels of four glucose metabolism markers known to play important roles during NAFLD progression were also analyzed. Similar patterns to those of lipid metabolism genes were observed for the triglyceride synthesis and glucose metabolism genes acyl coenzyme A (CoA): diacylglycerol acyltransferase 1 (DGAT1), DGAT2, and carbohydrate response element-binding proteins chREBP-α and chREBP-β which are key participants in insulin responses (Figure 6D).

**Figure 7:**
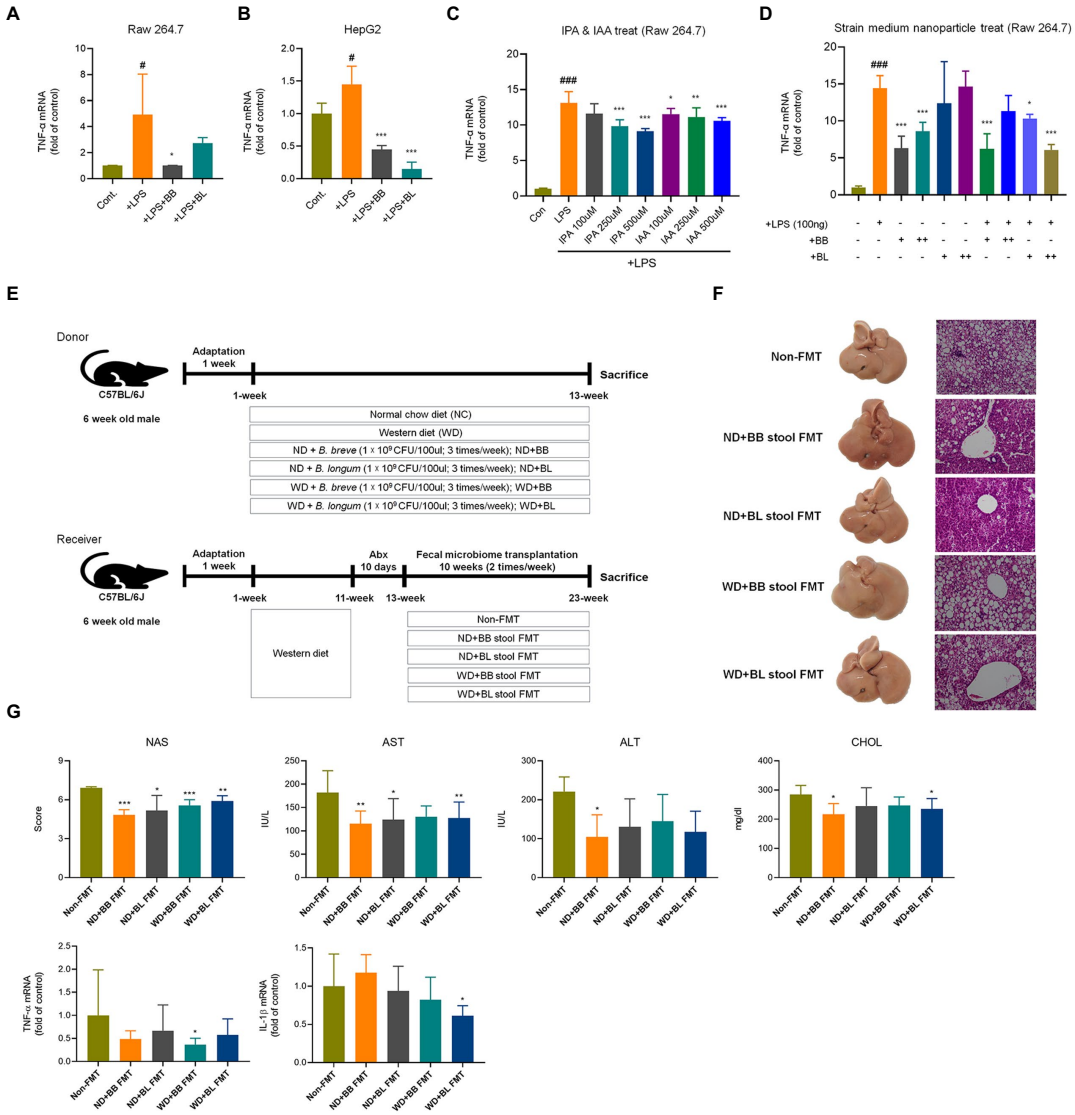
Comparison of anti-inflammatory effects of probiotic culture, indole metabolites, CFS nanoparticles, and fecal microbial transplantation **(A)** CFS on RAW 264.7 cells. **(B)** CFS on HepG2 cells. **(C)** IPA & IAA on RAW 264.7 cells, **(D)** CFS on RAW 264.7 cells. **(E)** Experiment design depicting the animal model used. **(F)** Representative liver specimens of gross examinations and H&E-stained liver sections. **(G)** NAS, and liver enzyme, and inflammatory cytokines. CFS, Cell free supernatant; BB, *Bifidobacterium breve* CFS; BL, *Bifidobacterium longum* CFS; TNF, Tumor necrosis factor; LPS, Lipopolysaccharides; IPA, 3-Indole propionic acid; IAA, Indole-3-acetic acid. # obtained statistics by comparing Cont. and LPS group. *statistics were obtained by comparing LPS group with the experimental group. One-way analysis of variance (ANOVA): ^#^*p* < 0.05, ^###^*p* < 0.001, ^*^p < 0.05, ^**^p < 0.01, and ^***^p < 0.001.

Additionally, the mRNA levels of inflammation markers and chemokines associated with immune cell infiltration were investigated. Cytokines and chemokines intervene in essential biological processes, such as inflammation and immunity, which are also associated with many pathologies, including NAFLD (Braunersreuther et al., 2012). In contrast to metabolic markers, the mRNA levels of the pro-inflammatory cytokines tumor necrosis factor (TNF)-α, interleukin (IL)-6 and IL-1β were not significantly different between the NC and WD groups (Figure 6E). The expression levels of IL-6 and IL-1β were found to be significantly lower in the BB and BL groups than in the WD group. In addition, the BL group showed significant downregulation of TNF-α. Since we could not observe significant differences in the mRNA levels of the above pro-inflammatory cytokines *in vivo*, we examined their effects *in vitro* using lipopolysaccharides (LPS) and indole metabolites as negative and positive controls, respectively. First, we incubated RAW 264.7 and HepG2 cells with bacterial cultures or LPS (positive control). Both strains significantly reduced the expression of TNF-α in both cell lines (Figures 7A,B). Next, we prepared a nanoparticle of cell free supernatant (CFS) of the probiotic strains and used 3-indole propionic acid (IPA) and indole-3-acetic acid (IAA) at different concentrations (100, 250, 500 μM) with or without LPS. As we expected, treatment with IPA and IAA significantly reduced TNF-α mRNA levels at all concentrations (Figure 7C). In a similar manner, treatment with *B. breve* and *B. longum* CFS-based nanoparticles downregulated TNF-α expression in a comparable manner (Figure 7D).

In the case of hepatic expression of chemokines, remarkable increases in mRNA levels of C–C motif chemokine ligand 2 (CCL2), CCL3, C-X-C motif chemokine ligand 9 (CXCL9) and CXCL16 were found in the WD group compared to those in the NC group (Figure 6F). These chemokines are known to induce cytotoxic T-cell recruitment to the liver (Oo et al., 2010). The chemokine CCL2 instigates inflammation in fat-accumulated tissues by facilitating the migration of inflammatory cells from the circulating blood (Arner et al., 2012). All four chemokines were significantly reduced in the probiotic supplement groups. In summary, probiotic treatments resulted in modulation of western diet-induced disturbances in hepatic metabolism, inflammation, and immune cell recruitment.

In the fecal microbial transplantation with BB and BL, NAS scores, liver enzyme, and cytokines were improved in BB and BL group (Figures 7E–G).

## Discussion

NAFLD comprises a broad array of liver pathogenesis ranging from simple steatosis to more severe complications (e.g., liver cirrhosis and hepatocellular carcinoma). A saturated fat-enriched western diet causes the development of NAFLD *via* lipid metabolic pathways in the liver (Cameron-Smith et al., 2003). One of the underlying mechanisms is a disrupting effect on gut microbiota. Alteration of gut microbiota is consequently involved in liver pathogenesis by disrupting gut barrier function and stimulating fat accumulation, inflammatory responses, and oxidative burden (Backhed et al., 2007). As gut microbiome research is still in its infancy, the literature provides inadequate data on the gut microbiota-liver axis. This limits progress in understanding the pathophysiological mechanisms and establishing targets for therapeutic strategies.

Accordingly, we investigated the effects of a western diet and evaluated the protective effects of two probiotic *Bifidobacterium* strains on the NAFLD progression. Indeed, the Western diet resulted in alteration in the gut microbiota and hepatic steatosis, thereby activating the pathophysiological mechanisms leading to NAFLD. The *Bifidobacterium* induced significant attenuation in regulating the gut microbiota, downregulating hepatic steatosis and inflammatory biomarkers, and improving liver function. Similarly, the intestinal metabolism group was treated by probiotic modulation. Gene expression related to lipid and glucose metabolism and immune responses suggests coordinated regulation of β-oxidation, lipid production and body inflammation by probiotic treatment.

Western diet challenge for 9 weeks resulted in an overall increase in body weight, liver weight, and liver size and consequently induced severe steatosis. The Western diet is mainly characterized by dietary intake of foods with higher saturated fat contents (Tilg and Moschen, 2015), and this study applied a mouse diet with 42% fat content. Our results showed that mice fed this Western diet exhibited higher steatosis scores, hepatitis scores and NASs, which indicated the development of pronounced NAFLD. Steatosis during a high-fat diet is caused by the availability of abundant saturated fat, which is responsible for intrahepatic triglyceride accumulation (Ress and Kaser, 2016). Several studies have reported that mice fed a Western diet develop NAFLD through weight gain and fat accumulation manifested by vacuolation of hepatocytes, accumulation of perilipin proteins, inflammation, and oxidative stress in the liver (Yang et al., 2020). The progression of NAFLD in this study was clearly revealed by the accumulation of microvesicular lipid droplets in the liver tissue, as shown by gross specimens of liver and H&E staining of liver tissue. Note the severe vacuolation of hepatocytes, which resulted in a strikingly whitish appearance of the liver of mice in the Western diet group. Treatment with the probiotic strains improved the above pathological indicators through a significant reduction in hepatic steatosis compared with the WD. A recent study reported that *B. lactis V9* attenuates NAFLD induced by a high-fat diet by mitigating hepatic steatosis (Yan et al., 2020). Our results showed that rapid development of a full-fledged chronic NAFLD pathology can be easily achieved in mice using a Western diet model. This is of paramount importance for the advancement of fundamental and preclinical therapeutic targeting studies on NAFLD, a disease that affects millions of people worldwide with an ever increasing trend (Wegermann et al., 2020).

To investigate whether the Western diet induces changes in gut microbiota, metagenomics analysis was conducted on fecal samples. At the phylum level, we noticed that the Western diet triggered a reduction in the relative abundance of *Bacteroidetes,* while it promoted an increase in *Proteobacteria* and *Firmicutes*. Among the most noticeable changes at the genus level was an increase in the relative abundance of *Helicobacter,* which is a member of *Proteobacteria*. While the mechanisms are still poorly understood, our results are in agreement with a previous study that reported the same trends in mice fed a high-fat diet (Hildebrandt et al., 2009). Previous studies have also confirmed that dysbiosis is linked to a high-fat diet and it plays important roles in the pathogenesis of NAFLD (Schnabl and Brenner, 2014). Dysbiosis due to a high-fat diet is suggested to be attributed to the creation of nutrient stress in the gut. For example, the lower proportion of carbohydrates in a high-fat diet is believed to cause a decrease in metabolism genes due to nutrient deficiency. Such conditions may enhance the overgrowth of certain bacterial taxa better suited for adapting to the environment while inhibiting others with selective pressure. It has been reported that *Bacteroidetes* are known to have large numbers of genes that encode carbohydrate-active enzymes, making them better suited to carbohydrate metabolism, while members of *Proteobacteria* are enhanced by a high-fat diet in the gut (Flint et al., 2012). Therefore, it is suggested that the high-fat content in the Western diet promoted overgrowth of *Proteobacteria* while inhibiting *Bacteroidetes.* Further analysis of bacterial and host metabolic enzyme patterns in the gut is required to determine such mechanisms.

Other important players in the gut microbiota and liver axis are microbe-derived metabolites. Some metabolites are synthesized by the microbes, and others are products of their enzymatic processes. We conducted metabolomic analysis of fecal microbe-derived metabolites, including SCFA, bile acids, and indole metabolites. Distinct metabolite profiles were observed among the different mouse groups. The reduction in SCFA levels in the WD group may indicate a decrease in the number and activity of bacteria capable of producing these metabolites. A previous study reported a decrease in fecal SCFA, such as acetate, propionate, and butyrate, in NAFLD patients with significant fibrosis, while no significant difference was observed for the moderate NAFLD stage (Rau et al., 2018). This trend was remarkably reversed in the probiotic *Bifidobacterium*-fed groups. A recent study demonstrated a decrease in *Bifidobacterium* and *Lactobacillus* in NAFLD patients (Niccolai et al., 2019). Therefore, the ameliorating effects of probiotic *Bifidobacterium* observed in this study can be attributed to their SCFA-producing ability. SCFAs are well known to inhibit hepatic cholesterol and lipogenesis while activating hepatic lipid oxidation (den Besten et al., 2013). Some indole derivatives, including methyl indole-3-acetic acid, indole-3-propioic acid, indole-3-acetic acid, 5-hydroxyindole-3-acetic acid, and indole-2-carboxylic acid, showed noticeable reduction in the WD group. This shows that the dysbiosis induced by the Western diet resulted in altered tryptophan metabolism. This is in agreement with a recent study that demonstrated a reduction in intestinal indole derivatives during dysbiosis of alcoholic liver disease in humans as well as experimental rodent models (Hendrikx and Schnabl, 2019). Indole derivatives alleviate hepatic steatosis and inflammation mainly by enhancing intestinal tight junctions and regulating intestinal immune homeostasis. For instance, some indole derivatives serve as ligands for the aryl hydrocarbon receptor, which is expressed by immune cells in the lamina propria and involved in pathogen defense through IL-22 expression (Ma et al., 2020). It also appears that the Western diet induced elevation of conjugated bile acids in this study. Bile acids undergo extensive microbe-mediated metabolism in the gut and are well known to greatly influence hepatic lipid accumulation. Probiotic supplementation remarkably reduced conjugated bile acid levels in the gut. Deconjugation is catalyzed by bacterial enzymes, primarily bile salt hydrolases, which are widespread in gut microorganisms, including *Bifidobacterium* and *Lactobacillus* (Rani et al., 2017).

To better understand the pathophysiological mechanisms of Western diet-related NAFLD at the molecular level, the mRNA levels of SREBP-1c, PPARα, and CCL2 genes in liver tissue were analyzed by qPCR. In the WD group, a marked elevation in hepatic mRNA of SREBP-1c was observed, while that of PPARα was lower than that in the control group, resulting in a higher SREBP-1c/PPARα ratio. The progression of NAFLD is mainly regulated by the expression of genes related to lipid metabolism. SREBP-1c plays a key role in the induction of lipogenesis in the liver, while PPARα favors fatty acid (FA) β-oxidation (Goto et al., 2013). The SREBP-1c/PPARα ratio has also been reported to be a good marker for determining the rate of hepatic steatosis (Pettinelli et al., 2009). The higher SREBP-1c/PPARα in the WD mice group was accompanied by an upregulation of CCL2, an inflammation initiator in fat-accumulated tissues (Arner et al., 2012). These results indicate that steatosis has led to pathologically considerable inflammation in the liver. In fact, a significant increase in the expression of IL-1β was observed, which suggests that it was induced by CCL2. CCL2 has been previously reported to induce significant secretion of several inflammatory cytokines, including IL-6 and IL-1β (Semple et al., 2010).

Randomized clinical trial studies have demonstrated that administration of probiotics attenuates NAFLD by alleviating hepatic steatosis and reducing hepatic inflammation (Ahn et al., 2019). Recent preclinical studies have reported amelioration of NAFLD by probiotic *Lactobacillus* and *Bifidobacterium* through modulation of gut microbiota-dependent pathways (Lee N. Y. et al., 2020). The results of the two *Bifidobacterium* strains used in this study are consistent with previous animal studies. *Bifidobacterium breve* and *B. longum* displayed efficient improvement of NAFLD by reducing liver weight, modulating gut microbiota, alleviating hepatic steatosis, and lowering inflammatory signaling molecules in the liver. According to the metagenomics data, the most noticeable gut microbes among the WD and probiotic treatment groups were *Bacteroidetes* and *Firmicutes.* These bacteria have been reported to be essential participants in host energy metabolism. *Firmicutes* are rich in genes involved in lipid digestion and nutrient movements, while *Bacteroidetes* have a lower capability to release extra energy from fat. Probiotic treatment with *Bifidobacterium* strains significantly increased the relative abundance of *Bacteroidetes.* This resulted in reduced mRNA levels of SREBP-1c (lipogenesis inducer) and CCL2 (inflammation initiator), while an upregulation of PPARα (inducer of β-oxidation) was observed compared with that in the WD group. Therefore, the attenuating effects of these strains on the overall NAFLD pathogenesis are mainly associated with their modulatory effect on the gut microbiota, resulting in reduced release of extra energy from fat, less triglyceride accumulation, and an inflammatory response. Of note, WD resulted in a significant increase in *Proteobacteria,* especially the *Helicobacter* genus. The relative abundances of *Helicobacter* tended to show a slight reduction in the probiotic treatment groups. Members of this genus are known to induce the development of acute and chronic inflammation in the intestine (Blosse et al., 2018). Considering that the gut microbiota is a potential driver of liver inflammation (Chassaing et al., 2014), it can be concluded that *Bifidobacterium* suppresses the inflammatory response. Compared with the WD group, significant reductions in the mRNA levels of the pro-inflammatory cytokines TNF-α, IL-6 and IL-1β were observed in the probiotic treatment groups.

In addition to the above pathological indicators of NAFLD, liver function was evaluated by measuring serum levels of AST, ALT, TBIL, and total CHOL. Serum levels of AST, ALT, TBIL, and total CHOL were markedly reduced in the probiotic *B. breve* and *B. longum* treatment groups compared to those in the WD group. All liver function test results showed a positive correlation with the biomarkers of NAFLD, indicating that liver injury can be prevented by alleviating the progression of NAFLD.

In summary, our results show that WD induced significant changes in microbial composition and resulted in development of hepatic steatosis as well as activation of inflammatory pathways. Treatment with *B. breve* and *B. longum* attenuated NAFLD by modulating the gut microbiota, downregulating hepatic steatosis and inflammation, and improving liver function. We suggest that these strains have the potential to be applied in the treatment of NAFLD patients.

## Materials and methods

### Probiotic strains

Two *Bifidobacterium* species namely *B. breve* CKDB002 and *B. longum* CKDB004 were used as probiotic strains in this study. These strains were originally isolated from feces of newborns and were obtained from Chong Kun Dang bioCorp (Gyeonggi-do, Korea) as processed lyophilized powder preparations.

### Patients

A total of 32 patients with NAFLD and 25 healthy subjects from Hallym University hospital (Admitted in from 2017/03 to 2021/03) were randomly recruited for the fecal microbial composition analysis (ClinicalTrials.gov NCT04339725). Patients with elevated liver enzyme [aspartate aminotransferase (AST) or alanine aminotransferase (ALT) ≥ 50 IU/L] were included in the hepatitis group. Enrolled patients for NAFLD who did not drink excessive alcohol and other liver diseases were excluded. Patients with viral hepatitis, autoimmune hepatitis, pancreatitis, hemochromatosis, Wilson’s disease, drug-induced liver injury, and other cancers were excluded. The eligibility criteria were based on age (40–60), NAFLD stage (hepatic steatosis-hepatitis), and body mass index (healthy subjects BMI ≦ 23 and NAFLD patients BMI > 23).

Baseline studies included family history, diet pattern, alcohol history, abdominal ultrasound, and computed tomography scan, X-ray, electrocardiography, complete blood count, electrolytes, liver function test, and viral markers. This project followed the ethics at 1975 Helsinki Declaration, as reflected by a prior approval by the institutional review board for human research in hospitals (2016-134). Informed consent was obtained from all participants.

### Animal experiments

Six weeks of age specific-pathogen free male C57BL/6 J mice were purchased from DooYeol Biotech (Seoul, Korea). Animals were housed at 22°C under controlled conditions with a 12-h: 12-h light/dark cycle and relative humidity of 55 ± 10%. During the 1-week adaptation period, mice had free access to normal chow diet and sterile water. After 1 week of acclimatization, mice were randomly divided into four different diet groups as follows. Normal chow diet group; 18% protein rodent diet (2018S TD, Envigo), WD group; rodent diet with 42% fat, 42.7% carbohydrate, 15% protein (TD88137, DooYeol Biotech), Probiotic administration groups *B. breve* CKDB002 and *B. longum* CKDB004; Provided with distilled water containing probiotic strains at 10^9^ CFU/g. After 9 weeks of treatment, animals were sacrificed after inhalation of anesthesia isoflurane. Body and liver weights were recorded. Whole blood samples were centrifuged at 19,000 ×*g* to collect serum. Liver, stool, and intestine samples were excised and immediately stored at −80°C.

The animals received humane care and all procedures were performed in accordance with National Institutes of Health Guidelines for the Care and Use of Laboratory Animals. All procedures were approved by the Institutional Animal Care and Use Committee of the College of Medicine, Hallym University (Hallym 2019-30).

### Chemicals and reagents

Normal rodent diet (2018S TD, Envigo) and WD (TD88137, DooYeol Biotech) were purchased from commercial suppliers, respectively. Lipopolysaccharide (LPS), 3-Indolepropionic acid (IPA), and Indole-3-acetic acid (IAA) were purchased from Sigma-Aldrich (St. Louis, MO, United States). HPLC grade methanol, acetonitrile, and deionized water were purchased from J.T. Baker Co. (Phillipsburg, NJ, United States). All the other reagents were of analytical grade.

### Histopathological examinations

Specimens were fixed with 10% formalin for 24 h, embedded in paraffin and tissue sections were cut for hematoxylin and eosin (H&E) staining analysis. The images of H&E-stained section were taken using a fluorescence microscope. Fatty liver was classified as according to NASH clinical research network scoring system for NAFLD from grades 0 to 3 (0: <5%, 1: 5%~33%, 2: 34%~66%, 3: >66% of steatosis). Inflammation was classified from grades 0 to 3 (0: none, 1: 1~2 foci per ×20 field, 2: 2~4 foci per ×20 field, and 3: >4 foci per ×20 field). All biopsy specimens were analyzed by a pathologist (S. H. H.). The NAFLD activity score (NAS), an objective index for classifying the grade of fatty liver, is suggested by Kleiner which is sum of the scores of diabetes, bovine inflammation, and balloon dilatation (Kleiner et al., 2005). According to the guidelines, NAS can help us recognize a histological scoring system addressing the full spectrum of NAFLD (Brunt et al., 1999). For statistical analyses, the patients were grouped into the three different NAS groups (group 1 = NAS 0–2: probable no NASH; group 2 = NAS 3–4: borderline; group 3 = NAS 5–8: probable NASH).

### Quantitative real time-polymerase chain reaction

Liver tissue samples stored at −80°C were homogenized in 1 mL TRIzol reagent (Invitrogen, Gaithersburg, MD, United States) and the total mRNA was isolated in accordance with the manufacturer’s instructions. Synthesis of cDNA was performed using the High-Capacity cDNA Reverse Transcription Kit (Applied Biosystems, Foster City, CA, United States) with random primers. The house-keeping gene GAPDH was used as an internal control to analyze the mRNA levels of TNF-α, IL-1β and IL-6. cDNA was amplified for quantitative real time PCR with One Step real-time PCR system (Applied Biosystems, Forster City, CA, United States) using PowerUp SYBR Green PCR Master Mix (Applied Biosystems, Foster City, CA, United States) and primer pairs (GenoTech, Daejeon, Korea). PCR primers were designed based on cDNA sequences from GenBank and were BLAST searched for specificity. Primers used in this study were as follows: GAPDH, forward 5′-AAATGGGGTGAGGCCGGT-3′ and reverse 5′-ATTGCTGACAATCTTGAGTGA-3′; TNF-α, forward 5′-CTGTAGCCCACGTCGTAGC-3′ and reverse 5′-TTGAGATCCATGCCGTTG-3′; IL-1β, forward 5′-TGTAATGAAAGACGGCACACC-3′ and reverse 5′-TCTTCTTTGGGTATTGCTTGG-3′; IL-6, forward 5′-CCACTTCACAAGTCGGAGGCTTA-3′ and reverse 5′-CCAGTTTGGTAGCATCCATCATTTC-3′. In qRT-PCR, the quantity of cDNA was calculated using the ΔΔCt method.

### Western blotting analysis

Western blot analysis was conducted as described previously (Nonoguchi et al., 1995). Total protein was isolated from the mouse intestine. Equal amounts of total protein were separated on a 12% SDS-polyacrylamide gels (SDS-PAGE) and transferred on to a nitrocellulose membrane. Membrane was blocked overnight in Tris-buffered saline (TBS) containing 0.05% Tween (TBST) and 5% dry powdered milk and then washed three times for 5 min each with TBST and incubated for 2 h at room temperature in primary antibody (rabbit anti-occludin, Sigma). After three washes with TBST, the membranes were incubated for 1 h with horseradish peroxidase-conjugated secondary antibody. Following two washes with TBST and one wash with TBS Blots were developed using the Enhanced Chemiluminescence (ECL) Western blotting detection reagents (Amersham-Pharmacia Biotech) and utilizing image capturing software (Amersham-Imager 680, version. 2.0.).

### *In vitro* assays

RAW 264.7, widely used as murine macrophage cell lines and HepG2 cells obtained from the Korean Cell Line Bank (KCLB) were used for the *in vitro* experiments. Cells were grown in Dulbecco’s Modified Eagle’s Medium (DMEM, Gibco BRL). For the stimulation and treatment assays, cells were plated at 3 × 10^5^ cells/well on 12 well plate with DMEM media. After 24 h of incubation, bacterial suspension, LPS, and CFS-based nanoparticles were added. After 12 h of incubation, cells were harvested for qRT-PCR analysis. For the indole metabolites treatment experiments, cells were pre-treated with IPA and IAA for 6 h followed by addition of LPS. Subsequently, cells were harvested for total RNA isolation after 12 h of incubation.

### Trans-epithelial electrical resistance measurements

Caco-2 cells were seeded onto Transwell-Clear inserts (12-well clusters, 6.5-mm inserts with polyester membrane, pore diameter 0.4 μm, Corning NY) at a density of 10^5^ cells/insert. Each insert was placed on top of a well in a 24-well plate with 1 ml in the bottom and 200 μL media in the top as described previously (Anderson et al., 2010). Caco-2 cells were grown for 5 days until confluence in Minimum Essential Medium Eagle (MEM) with 20% fetal bovine serum (FBS) without antibiotic-antimycotic (Gibco, Carlsbad, CA, United States) at 37°C in a humidified 5% atmosphere. TEER measurements were performed using a Millicell Electrical resistance system (Millipore, Billerica, MA, United States). When monolayer of cells reached the confluence, Caco-2 cells were co-incubated with 200 μL of bacterial culture grown to OD600 0.3 (7 × 10^7^ CFU/mL) in MEM media. Consequently, the TEER was measured after 8 h of incubation.

### Serum analysis

AST, ALT, TBIL, and CHOL were determined using a commercial biochemical analyzer of blood (KoneLab 20, Thermo Fisher Scientific, Waltham, Finland).

### Statistical analysis

Continuous variables were expressed as means and standard deviations. One-way ANOVA and independent sample *t*-test were performed during the liver and body weight, L/B ratio, liver function test, and histopathological analyses. All statistical analyses were done using IBM SPSS statistics program (IBM software, Armonk, NY, United States). Any values lying below *p* < 0.05 were considered statistically significant. Results were represented as mean ± standard deviation.

### Bioinformatics

Statistical analyses were conducted on all continuous variables acquired from GC-MS and LC-MS. All datasets were normalized using the “MS total useful signal” (Li et al., 2017). Significant differences between two groups were determined by Mann–Whitney *U*-test and Student’s *t*-test. A Kruskal-Wallis test with Dunn’s *post hoc* was conducted to evaluate significant differences among four groups using package Dunn’s Test in the software R (Dinno and Dinno, 2017). The p-value was corrected by Benjamini-hochberg’s adjustment (false discovery rate) and pathway over-representation analysis were performed based on the statistical modules implemented in MetaboAnalyst 4.0 (based on the hypergeometric test and relative-betweenness centrality; Chong et al., 2018). Treemap and Pie chart were created through Microsoft Excel (Microsoft, Seattle, WA, United States) using compound classification by Human Metabolome Database (Wishart et al., 2018). The metabolic network map was constructed based on structural similarity (Tanimoto score) and biochemical liaison (KEGG reaction pair information), and visualized by a prefuse force-directed layout using Cytoscape version 3.7.2 (Shannon et al., 2003). SIMCA 15 (Umetrics AB, Umea, Sweden) was applied for multivariate statistics including principal component analysis. Heat map, Column scatter graph, Violin plot, and Volcano plot were generated using GraphPad prism software ver. 7 (GraphPad Software Inc., San Diego, CA, USA). Co-inertia analysis was performed in the M2IA server^1^ (Ni et al., 2020). Interomic correlation matrix between individual metabolite and microbial composition (at genus level) was constructed based on Spearman’s rank analysis (package stats in the software R).

## Data availability statement

The datasets presented in this study can be found in online repositories. The names of the repository/repositories and accession number(s) can be found in the article/Supplementary material.

## Ethics statement

This project followed the ethics at 1975 Helsinki Declaration, as reflected by a prior approval by the institutional review board for human research in hospitals (2016-134). Informed consent was obtained from all participants. The patients/participants provided their written informed consent to participate in this study. All procedures were approved by the Institutional Animal Care and Use Committee of the College of Medicine, Hallym University (Hallym 2019-30).

## Author contributions

SY, JY, DL, and KS designed the study and interpreted the work. SY and JY wrote the manuscript. BM, HG, S-MW, HP, SH, B-YK, KK, BK, HJ, and T-SP performed experiments. SH provided tissue specimens. All authors contributed to the article and approved the submitted version.

## Funding

This research was supported by Hallym University Research Fund, Korea National Research Foundation (2020R1A6A1A03043026 and 2021M3A9I4021433), Bio Industrial Technology Development Program (20018494) funded by the Ministry of Trade, Industry and Energy (MOTIE, Korea), the Promotion of Innovative Businesses for Regulation-Free Special Zones funded by the Ministry of SMEs and Startups (MSS, Korea; P0020622), and The Korea Institute of Planning and Evaluation for Technology in Food, Agriculture and Forestry (IPET) through High Value-added Food Technology Development Program, funded by Ministry of Agriculture, Food and Rural Affairs (MAFRA; 321036-05-1-HD020).

## Conflict of interest

The authors declare that the research was conducted in the absence of any commercial or financial relationships that could be construed as a potential conflict of interest.

## Publisher’s note

All claims expressed in this article are solely those of the authors and do not necessarily represent those of their affiliated organizations, or those of the publisher, the editors and the reviewers. Any product that may be evaluated in this article, or claim that may be made by its manufacturer, is not guaranteed or endorsed by the publisher.

## Supplementary material

The Supplementary material for this article can be found online at: https://www.frontiersin.org/articles/10.3389/fmicb.2023.1129904/full#supplementary-material

## Footnotes

^1^ http://m2ia.met-bioinformatics.cn/

## References

[ref1] Adams L. A. Angulo P. (2005). Recent concepts in non-alcoholic fatty liver disease. Diabet. Med. 22, 1129–1133. doi: 10.1111/j.1464-5491.2005.01748.x 16108837

[ref2] Ahn S. B. Jun D. W. Kang B. K. Lim J. H. Lim S. Chung M. J. (2019). Randomized, double-blind, placebo-controlled study of a multispecies probiotic mixture in nonalcoholic fatty liver disease. Sci. Rep. 9:5688. doi: 10.1038/s41598-019-42059-3, PMID: 30952918 PMC6450966

[ref3] Anderson R. C. Cookson A. L. Mcnabb W. C. Park Z. Mccann M. J. Kelly W. J.. (2010). Lactobacillus plantarum Mb452 enhances the function of the intestinal barrier by increasing the expression levels of genes involved in tight junction formation. BMC Microbiol. 10:316. doi: 10.1186/1471-2180-10-316, PMID: 21143932 PMC3004893

[ref4] Arner E. Mejhert N. Kulyte A. Balwierz P. J. Pachkov M. Cormont M.. (2012). Adipose tissue micrornas as regulators of Ccl2 production in human obesity. Diabetes 61, 1986–1993. doi: 10.2337/db11-1508, PMID: 22688341 PMC3402332

[ref5] Backhed F. Manchester J. K. Semenkovich C. F. Gordon J. I. (2007). Mechanisms underlying the resistance to diet-induced obesity in germ-free mice. Proc. Natl. Acad. Sci. U. S. A. 104, 979–984. doi: 10.1073/pnas.0605374104, PMID: 17210919 PMC1764762

[ref6] Blosse A. Lehours P. Wilson K. T. Gobert A. P. (2018). Helicobacter: inflammation, immunology, and vaccines. Helicobacter 23:e12517. doi: 10.1111/hel.12517, PMID: 30277626 PMC6310010

[ref7] Braunersreuther V. Viviani G. L. Mach F. Montecucco F. (2012). Role of cytokines and chemokines in non-alcoholic fatty liver disease. World J Gastroenterol: WJG 18, 727–735. doi: 10.3748/wjg.v18.i8.727, PMID: 22371632 PMC3286135

[ref8] Brunt E. M. Janney C. G. Di Bisceglie A. M. Neuschwander-Tetri B. A. Bacon B. R. (1999). Nonalcoholic steatohepatitis: a proposal for grading and staging the histological lesions. Am. J. Gastroenterol. 94, 2467–2474. doi: 10.1111/j.1572-0241.1999.01377.x, PMID: 10484010

[ref9] Cameron-Smith D. Burke L. M. Angus D. J. Tunstall R. J. Cox G. R. Bonen A.. (2003). A short-term, high-fat diet up-regulates lipid metabolism and gene expression in human skeletal muscle. Am. J. Clin. Nutr. 77, 313–318. doi: 10.1093/ajcn/77.2.313, PMID: 12540388

[ref10] Chassaing B. Etienne-Mesmin L. Gewirtz A. T. (2014). Microbiota-liver axis in hepatic disease. Hepatology 59, 328–339. doi: 10.1002/hep.26494, PMID: 23703735 PMC4084781

[ref11] Chong J. Soufan O. Li C. Caraus I. Li S. Bourque G.. (2018). MetaboAnalyst 4.0: towards more transparent and integrative metabolomics analysis. Nucleic Acids Res. 46, W486–W494. doi: 10.1093/nar/gky310, PMID: 29762782 PMC6030889

[ref12] Cohen J. C. Horton J. D. Hobbs H. H. (2011). Human fatty liver disease: old questions and new insights. Science 332, 1519–1523. doi: 10.1126/science.1204265, PMID: 21700865 PMC3229276

[ref13] Debes J. D. Boonstra A. De Knegt R. J. (2020). Nafld-related hepatocellular carcinoma and the four horsemen of the apocalypse. Hepatology 71, 774–776. doi: 10.1002/hep.31170, PMID: 32039490 PMC7135940

[ref14] Den Besten G. Van Eunen K. Groen A. K. Venema K. Reijngoud D. J. Bakker B. M. (2013). The role of short-chain fatty acids in the interplay between diet, gut microbiota, and host energy metabolism. J. Lipid Res. 54, 2325–2340. doi: 10.1194/jlr.R036012, PMID: 23821742 PMC3735932

[ref15] Dinno A. Dinno M. A. (2017). Package ‘dunn. test’. Cran Repository, 10. Available at: https://CRAN.R-project.org/package=dunn.test

[ref16] Flint H. J. Scott K. P. Duncan S. H. Louis P. Forano E. (2012). Microbial degradation of complex carbohydrates in the gut. Gut Microbes 3, 289–306. doi: 10.4161/gmic.19897, PMID: 22572875 PMC3463488

[ref17] Gastaldelli A. Kozakova M. Hújlund K. Flyvbjerg A. Favuzzi A. Mitrakou A.. (2009). Fatty liver is associated with insulin resistance, risk of coronary heart disease, and early atherosclerosis in a large European population. Hepatology 49, 1537–1544. doi: 10.1002/hep.22845, PMID: 19291789

[ref18] Goto T. Kim Y. I. Takahashi N. Kawada T. (2013). Natural compounds regulate energy metabolism by the modulating the activity of lipid-sensing nuclear receptors. Mol. Nutr. Food Res. 57, 20–33. doi: 10.1002/mnfr.201200522, PMID: 23180608

[ref19] Hendrikx T. Schnabl B. (2019). Indoles: metabolites produced by intestinal bacteria capable of controlling liver disease manifestation. J. Intern. Med. 286, 32–40. doi: 10.1111/joim.12892, PMID: 30873652

[ref20] Hildebrandt M. A. Hoffmann C. Sherrill-Mix S. A. Keilbaugh S. A. Hamady M. Chen Y. Y.. (2009). High-fat diet determines the composition of the murine gut microbiome independently of obesity. Gastroenterology 137, e1–e2. doi: 10.1053/j.gastro.2009.08.042 PMC2770164 19706296

[ref21] Kleiner D. E. Brunt E. M. Van Natta M. Behling C. Contos M. J. Cummings O. W.. (2005). Design and validation of a histological scoring system for nonalcoholic fatty liver disease. Hepatology 41, 1313–1321. doi: 10.1002/hep.20701 15915461

[ref22] Kleiner D. E. Makhlouf H. R. (2016). Histology of nonalcoholic fatty liver disease and nonalcoholic steatohepatitis in adults and children. Clin. Liver Dis. 20, 293–312. doi: 10.1016/j.cld.2015.10.011, PMID: 27063270 PMC4829204

[ref23] Lee B. M. Lee E. M. Kang D. J. Seo J.-A. Choi H.-K. Kim Y.-S.. (2020). Discovery study of integrative metabolic profiles of sesame seeds cultivated in different countries. LWT 129:109454. doi: 10.1016/j.lwt.2020.109454

[ref24] Lee N. Y. Yoon S. J. Han D. H. Gupta H. Youn G. S. Shin M. J.. (2020). Lactobacillus and Pediococcus ameliorate progression of non-alcoholic fatty liver disease through modulation of the gut microbiome. Gut Microbes 11, 882–899. doi: 10.1080/19490976.2020.1712984, PMID: 31965894 PMC7524267

[ref25] Leung C. Rivera L. Furness J. B. Angus P. W. (2016). The role of the gut microbiota in Nafld. Nat. Rev. Gastroenterol. Hepatol. 13, 412–425. doi: 10.1038/nrgastro.2016.85 27273168

[ref26] Li B. Tang J. Yang Q. Li S. Cui X. Li Y.. (2017). Noreva: normalization and evaluation of Ms-based metabolomics data. Nucleic Acids Res. 45, W162–W170. doi: 10.1093/nar/gkx449, PMID: 28525573 PMC5570188

[ref27] Ma L. Li H. Hu J. Zheng J. Zhou J. Botchlett R.. (2020). Indole alleviates diet-induced hepatic steatosis and inflammation in a manner involving myeloid cell 6-Phosphofructo-2-kinase/Fructose-2,6-Biphosphatase 3. Hepatology 72, 1191–1203. doi: 10.1002/hep.31115, PMID: 31953865 PMC7365739

[ref28] Malesza I. J. Malesza M. Walkowiak J. Mussin N. Walkowiak D. Aringazina R.. (2021). High-fat, Western-style diet, systemic inflammation, and gut microbiota: a narrative review. Cells 10:3164. doi: 10.3390/cells10113164 34831387 PMC8619527

[ref29] Milani C. Duranti S. Bottacini F. Casey E. Turroni F. Mahony J.. (2017). The first microbial colonizers of the human gut: composition, activities, and health implications of the infant gut microbiota. Microbiol. Mol. Biol. Rev. 81:e00036-17. doi: 10.1128/MMBR.00036-17, PMID: 29118049 PMC5706746

[ref30] Minami J. Iwabuchi N. Tanaka M. Yamauchi K. Xiao J. Z. Abe F.. (2018). Effects of Bifidobacterium breve B-3 on body fat reductions in pre-obese adults: a randomized, double-blind, placebo-controlled trial. Biosci. Microbiota Food Health 37, 67–75. doi: 10.12938/bmfh.18-001, PMID: 30094122 PMC6081611

[ref31] Ni Y. Yu G. Chen H. Deng Y. Wells P. M. Steves C. J.. (2020). M2ia: a web server for microbiome and metabolome integrative analysis. Bioinformatics 36, 3493–3498. doi: 10.1093/bioinformatics/btaa188, PMID: 32176258

[ref32] Niccolai E. Baldi S. Ricci F. Russo E. Nannini G. Menicatti M.. (2019). Evaluation and comparison of short-chain fatty acids composition in gut diseases. World J. Gastroenterol. 25, 5543–5558. doi: 10.3748/wjg.v25.i36.5543, PMID: 31576099 PMC6767983

[ref33] Nonoguchi H. Owada A. Kobayashi N. Takayama M. Terada Y. Koike J.. (1995). Immunohistochemical localization of V2 vasopressin receptor along the nephron and functional role of luminal V2 receptor in terminal inner medullary collecting ducts. J. Clin. Invest. 96, 1768–1778. doi: 10.1172/JCI118222, PMID: 7560068 PMC185813

[ref34] Oo Y. H. Shetty S. Adams D. H. (2010). The role of chemokines in the recruitment of lymphocytes to the liver. Dig. Dis. 28, 31–44. doi: 10.1159/000282062, PMID: 20460888 PMC2883834

[ref35] Pettinelli P. Del Pozo T. Araya J. Rodrigo R. Araya A. V. Smok G.. (2009). Enhancement in liver Srebp-1c/Ppar-alpha ratio and steatosis in obese patients: correlations with insulin resistance and n-3 long-chain polyunsaturated fatty acid depletion. Biochim. Biophys. Acta 1792, 1080–1086. doi: 10.1016/j.bbadis.2009.08.015, PMID: 19733654

[ref36] Rani R. P. Anandharaj M. Ravindran A. D. (2017). Characterization of bile salt hydrolase from lactobacillus gasseri Fr4 and demonstration of its substrate specificity and inhibitory mechanism using molecular docking analysis. Front. Microbiol. 8:1004. doi: 10.3389/fmicb.2017.01004, PMID: 28620369 PMC5449720

[ref37] Rau M. Rehman A. Dittrich M. Groen A. K. Hermanns H. M. Seyfried F.. (2018). Fecal Scfas and Scfa-producing bacteria in gut microbiome of human Nafld as a putative link to systemic T-cell activation and advanced disease. United Eur. Gastroenterol. J. 6, 1496–1507. doi: 10.1177/2050640618804444, PMID: 30574320 PMC6297934

[ref38] Ray A. Basu S. Gharaibeh R. Z. Cook L. C. Kumar R. Lefkowitz E. J.. (2015). Gut microbial Dysbiosis due to helicobacter drives an increase in marginal zone B cells in the absence of Il-10 signaling in macrophages. J. Immunol. 195, 3071–3085. doi: 10.4049/jimmunol.1500153, PMID: 26324769 PMC4575870

[ref39] Ress C. Kaser S. (2016). Mechanisms of intrahepatic triglyceride accumulation. World J. Gastroenterol. 22, 1664–1673. doi: 10.3748/wjg.v22.i4.1664, PMID: 26819531 PMC4721997

[ref40] Schnabl B. Brenner D. A. (2014). Interactions between the intestinal microbiome and liver diseases. Gastroenterology 146, 1513–1524. doi: 10.1053/j.gastro.2014.01.020, PMID: 24440671 PMC3996054

[ref41] Semple B. D. Frugier T. Morganti-Kossmann M. C. (2010). Ccl2 modulates cytokine production in cultured mouse astrocytes. J. Neuroinflammation 7:67. doi: 10.1186/1742-2094-7-67, PMID: 20942978 PMC2964657

[ref42] Shannon P. Markiel A. Ozier O. Baliga N. S. Wang J. T. Ramage D.. (2003). Cytoscape: a software environment for integrated models of biomolecular interaction networks. Genome Res. 13, 2498–2504. doi: 10.1101/gr.1239303, PMID: 14597658 PMC403769

[ref43] Tilg H. Moschen A. R. (2015). Food, immunity, and the microbiome. Gastroenterology 148, 1107–1119. doi: 10.1053/j.gastro.2014.12.036 25575570

[ref44] Wegermann K. Suzuki A. Mavis A. M. Abdelmalek M. F. Diehl A. M. Moylan C. A. (2020). Tackling Nafld: three targeted populations. Hepatology 73, 1199–1206. doi: 10.1002/hep.31533 32865242

[ref45] Wishart D. S. Feunang Y. D. Marcu A. Guo A. C. Liang K. Vazquez-Fresno R.. (2018). Hmdb 4.0: the human metabolome database for 2018. Nucleic Acids Res. 46, D608–D617. doi: 10.1093/nar/gkx1089, PMID: 29140435 PMC5753273

[ref46] Yan Y. Liu C. Zhao S. Wang X. Wang J. Zhang H.. (2020). Probiotic Bifidobacterium lactis V9 attenuates hepatic steatosis and inflammation in rats with non-alcoholic fatty liver disease. AMB Exp. 10:101. doi: 10.1186/s13568-020-01038-y, PMID: 32472368 PMC7260323

[ref47] Yang P. Wang Y. Tang W. Sun W. Ma Y. Lin S.. (2020). Western diet induces severe nonalcoholic steatohepatitis, ductular reaction, and hepatic fibrosis in liver Cgi-58 knockout mice. Sci. Rep. 10:4701. doi: 10.1038/s41598-020-61473-6, PMID: 32170127 PMC7070035

[ref48] Yin Y. N. Yu Q. F. Fu N. Liu X. W. Lu F. G. (2010). Effects of four Bifidobacteria on obesity in high-fat diet induced rats. World J. Gastroenterol. 16, 3394–3401. doi: 10.3748/wjg.v16.i27.3394, PMID: 20632441 PMC2904885

[ref49] Younossi Z. M. (2019). Non-alcoholic fatty liver disease—a global public health perspective. J. Hepatol. 70, 531–544. doi: 10.1016/j.jhep.2018.10.033, PMID: 30414863

[ref50] Younossi Z. M. Stepanova M. Negro F. Hallaji S. Younossi Y. Lam B.. (2012). Nonalcoholic fatty liver disease in lean individuals in the United States. Medicine 91, 319–327. doi: 10.1097/MD.0b013e3182779d49 23117851

