# Supplementary material for: Bifidobacterium-derived short-chain fatty acids and indole compounds attenuate nonalcoholic fatty liver disease by modulating gut-liver axis
Source: Front Microbiol. 2023 Mar 1;14:1129904. doi: 10.3389/fmicb.2023.1129904 (PMC10014915; doi:10.3389/fmicb.2023.1129904)
Supplement: Supplementary file 1 [file Data_Sheet_1.pdf]

Supplementary Material

Bifidobacterium-derived short chain fatty acids and indole compounds attenuate nonalcoholic fatty liver disease by modulating gut-liver axis

Sang Jun Yoon[1,†], Jeong Seok Yu[2,†], Byeong Hyun Min[1,†], Haripriya Gupta[1], Sung-Min Won[1], Hee Jin Park[1], Sang Hak Han[3], Byung-Yong Kim[4], Kyung Hwan Kim[5], Byoung Kook Kim[5], Hyun Chae Joung[5], Tae-Sik Park[6], Young Lim Ham[7], Do Yup Lee[2,*], Ki Tae Suk[1,*]

*** Correspondence: Ki Tae Suk, M.D., Ph.D. ktsuk@hallym.ac.kr; Do Yup Lee, Ph.D. rome73@snu.ac.kr**

Supplementary Data

1. Fecal metagenomics analysis

Genomic DNA was extracted from stool samples using QIAamp stool kit (cat. no. 51504) as per the manufacturer's instructions. V3 - V4 region of the bacterial 16S rRNA gene was amplified using barcoded universal primers according to the following PCR conditions: an initial denaturation at 95 °C for 5 min, 20 cycles of 95 °C for 30 seconds, 55 °C for 30 seconds, and 72 °C for 30 seconds, followed by a final extension at 72 °C for 10 min. Purification of the amplicons was conducted with an Agencourt AMPure XP system (Beckman, USA) and quantification of the purified amplicons was conducted using PicoGreen and quantitative PCR. After pooling of the barcoded amplicons, sequencing was carried out using a MiSeq sequencer on the Illumina platform (ChunLab Inc., Republic of Korea) according to the manufacturer's specification.

Microbiome profiling was conducted with the 16S-based Microbial Taxonomic Profiling (MTP) platform of EzBioCloud Apps (ChunLab Inc., Republic of Korea). After taxonomic profiling of each sample, comparative MTP analyzer of EzBioCloud Apps was used for comparative analysis of the samples. In the MTP platform of ChunLab, preprocessing of the sequencing reads was conducted using the following five steps: 1) filtering of low-quality reads 2) merging of the paired-end reads, 3) removal of barcode and primer

sequences 4) taxonomic assignment of the reads and 5) removal of chimeric sequences. Taxonomic assignment of the reads was conducted with ChunLab's 16S rRNA database (DB ver. PKSSU4.0)[52]. OTU picking was conducted with UCLUST and CDHIT with 97% of the similarity cutoff[53]. Then, Good's coverage, rarefaction, and alpha-diversity indices, including ACE, Chao1, Jackknife, Shannon, Simpson, and NPShannon were calculated. Beta-diversity including PCoA and UPGMA clustering was shown in the comparative MTP analyzer. All 16S rRNA sequences were deposited in the ChunLab's EzBioCloud Microbiome database and sequencing reads of the 16S rRNA gene from this study were deposited in the NCBI Short Read Archive under the bioproject number PRJNA532302.

2. *Metabolic profiling*

The metabolic profiles of mouse cecal samples were obtained by gas chromatography–mass spectrometry (GC-MS) and liquid chromatography (LC)-MS. All of the mouse cecum samples were thawed under ice and mixed with 1,100 µl of extraction solvent A (1:1 v/v of acetonitrile/water). The mixtures were vortexed vigorously until the cecum was uniformly suspended and centrifuge for 5 min at 13,200 rpm. The supernatants (500 µl) were dispensed into the new tube (2 ml) for short chain fatty acids analysis (method 1). And then, extraction solvent B (1:3 v/v of acetonitrile/methanol) was added to the rest part of the supernatant. The mixtures were vortexed for 1 min to proceed with the secondary extraction, followed by centrifugation at 13,200 rpm for 5 minutes. The supernatant (500 µl) was transferred to a new tube (1.5 ml) for liquid chromatography Orbitrap mass spectrometry (method 2) and gas chromatography time-of-flight mass spectrometry (method 3), respectively. The supernatant was completely concentrated using a centrifugal vacuum concentrator (SCANVAC, Korea).

Method 1: Targeted analysis for short chain fatty acids using LC-MS

The supernatant (40 µl) dispensed from first extraction process was mixed with 20 µl of an EDC (1-ethyl-3-(3-dimethylaminopropyl) carbodiimide) –HCL at 120mM concentration in 6 % pyridine solution and 20 µl of

a 3NPH (mM 3-nitrophenylhydrazine) –HCL dissolved in 70 % acetonitrile. The mixture was reacted at 40 °C for 30 minutes, and then mixed with 1.92 ml of 70% acetonitrile (ref)

The derivatives were analysed by Thermo Q-Exactive Plus Orbitrap combined with an Ultimate-3000 UPLC system. Chromatographic separation was conducted through 2.1 x 150 mm ACQUITY BEH C18 with 1.7-μm particles column equipped with 5.0 x 2.1 mm BEH C18 VanGuard Pre-Column. The mobile phase included water (0.01 % formic acid) and acetonitrile (0.01 % formic acid). The gradient of LC was pre-set as follows:

Equilibration in 15% buffer B for 2 min, 15-55 % buffer B gradient over 9 min, 100 % buffer B held for 1min and re-equilibration in 15 % buffer B for 3 min. Injection volume was 2 μl for both MS1 and MS/MS analysis. Mass spectra were acquisition using Q-Exactive Plus Orbitrap (Thermo Fisher Scientific, Waltham, MA, USA) equipped with an electrospray ionization (ESI) interface (HESI-II) in negative ionization, and the system was controlled using Xcalibur 4.0 and Q-Exactive Tune software.

Raw data were processed by Tracefinder software (version 4.0, Thermo Fisher Scientific, San José, CA, USA). A mass tolerance for precursor ion and retention time tolerance were set to 5 ppm and 0.5 min, respectively

Method 2: Primary metabolites profiling using GC-TOF MS

The dried metabolites were derivatized with 5 μl of 40 mg/ml methoxyamine hydrochloride (Sigma-Aldrich, St. Louis, MO, USA) dissolved in pyridine (Sigma-Aldrich, St. Louise, MO, USA) for 90 min at 30 °C. After the first derivatization step, The second derivatization step was done with 45 μl of N-methyl-N-trimethylsilyltrifluoroacetamide (MSTFA +1% TMCS; Restek, Bellefonte, PA, USA) for 1 hour for 60 min at 37°C. Internal retention time index was added which included 13 fatty acids methyl esters (C8, C9, C10, C12, C14, C16, C18, C20, C22, C24, C26, C28, and C30).[54]

The injection (5 μl) of the derivatized metabolites was programmed by and an Agilent 7693 ALS (Agilent Technologies, Wilmington, DE, USA) in splitless mode. GC-TOF MS analysis was performed using an Agilent 7890B gas chromatograph (Agilent Technologies) and Leco Pegasus HT time of flight mass

spectrometer (LECO, St. Joseph, MI, USA). Oven temperature was initiated at 50°C (1 min), gradually increased at 20°C/min to 330°C, and held constant for 5 min. Transfer line and ion source temperatures were set to 280°C and 250°C, respectively. Mass spectra were collected ranging from 85 to 500 m/z at a scan rate of 20 spectra/sec with a detector voltage of 1850 V.[55]

Data pre-processing was conducted using ChromaTOF software (version 4.5), which included apex mass values, full spectrum, peak purity, signal-to-noise ratio, and retention time. Generic text file (.txt) and netCDF file were produced based on ChromaTOF-specific Pegasus file (.peg) for peak identification and semi-quantification. The post-process was performed using *Binbase* algorithm including chromatogram validation, primary RI detection, and validation of unique mass.[56, 57] A peak height of single quant ion was generated for statistical analysis. Missing values that did not pass the primary criteria were imputed by post-matching and replacement using raw data as previously described [54]. To evaluate analytical precision, a mixture of 25 pure reference compounds were analyzed every 6 samples, which demonstrated reproducibility during analysis (supplementary figure 7).

Method 3: secondary metabolites profiling of LC-Orbitrap MS

The dried extracts were reconstituted with 50 µl of 70% acetonitrile for LC-Orbitrap MS analysis. Chromatographic separation was carried out using Ultmate-3000 UPLC system (Thermo Fisher Scientific, Waltham, MA, USA) and a 150 × 2.1 mm UPLC BEH 1.7µm C18 column (Waters, Milford, MA, USA) equipped with 5.0 mm × 2.1 mm UPLC BEH 1.7µm C18 VanGuard Pre-Column (Waters, Milford, MA, USA). Mobile phase consisted of buffer A (0.1% formic acid in water) and buffer B (0.1% formic acid in 100% acetonitrile). A flow rate was set to 0.35 ml/min and a gradient was programmed as follows: equilibration in 3% buffer B for 1 min, 3-100% buffer B gradient over 9 min, 100% buffer B held for 1 min and re-equilibration in 3% buffer B for 3 min.

Mass-spectrometric analysis was performed on a Q-Exactive plus Orbitrap (Thermo Fisher Scientific, Waltham, MA, USA) with ionization polarity-switching mode. Full MS scan was conducted on the metabolites (70-1000 m/z) with resolution of 70,000 FWHM at m/z=200 and with automatic gain control

(AGC) target of 1e6 ions and maximum injection time (IT) of 100 ms. Data-dependent MS/MS analysis was performed on pooled samples by each ionization mode. Data-dependent MS/MS setting was as follows: Top10 MS1 ions; resolution, 17,500 at 200 m/z; AGC target, 1e5; maximum IT, 50 ms; isolation window, 1.0m/z; normalized collision energy (NCE), 30; intensity threshold, 2e3 ions; apex trigger, 3-6 sec; dynamic exclusion, 6 sec. M/Z values and retention times for 7 bile acids were added to the inclusion list for the application of higher collision energy (NCE, 70).

Data acquisition and pre-processing were conducted using Xcalibur software (Thermo Fisher Scientific, San José, CA, USA). The obtained RAW data files were processed using Compound Discoverer software (version 2.0, Thermo Fisher Scientific, San José, CA, USA). The data processing was done following the workflow such as Select spectra, Align Retention times, Detect Unknown Compounds, Group Unknown Compounds, Fill Gaps and Search mzCloud. Mass tolerance of MS1 on every node was set at 5 ppm. Align Retention Time node was set to 1 min to Maximum shift. Compound identification was done against mzCloud with criteria of 10 ppm (MS2 mass tolerance) and 70% of assignment threshold.

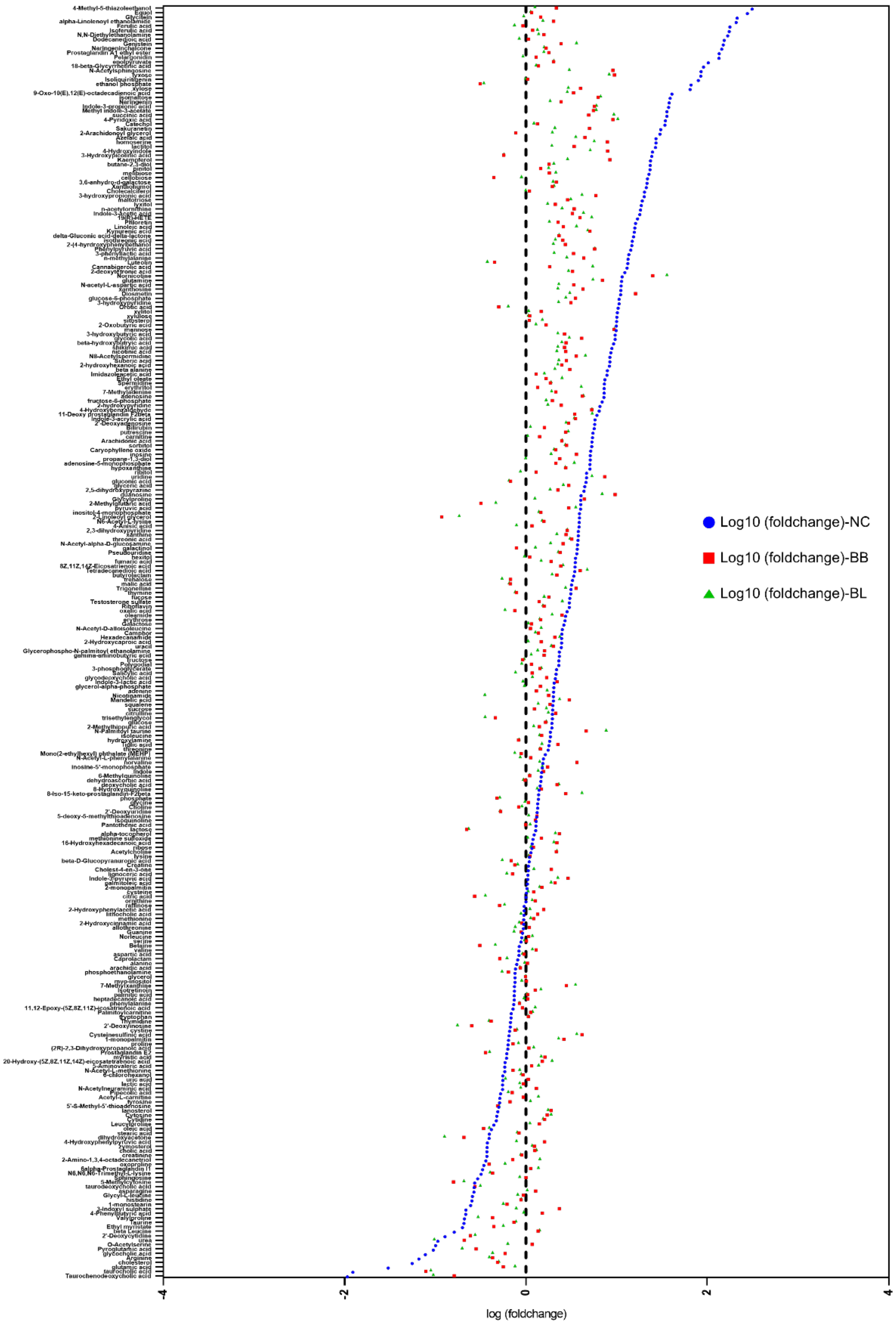

SUPPLEMENTARY FIGURE 1: Enrichment analysis demonstrating chemical class-wise quantitative

features of NC, BB, and BL groups, respectively compared to the WD group. NC, Normal chow diet group; WD, Western diet group; BB, *B. breve* group; BL, *B. longum* group.

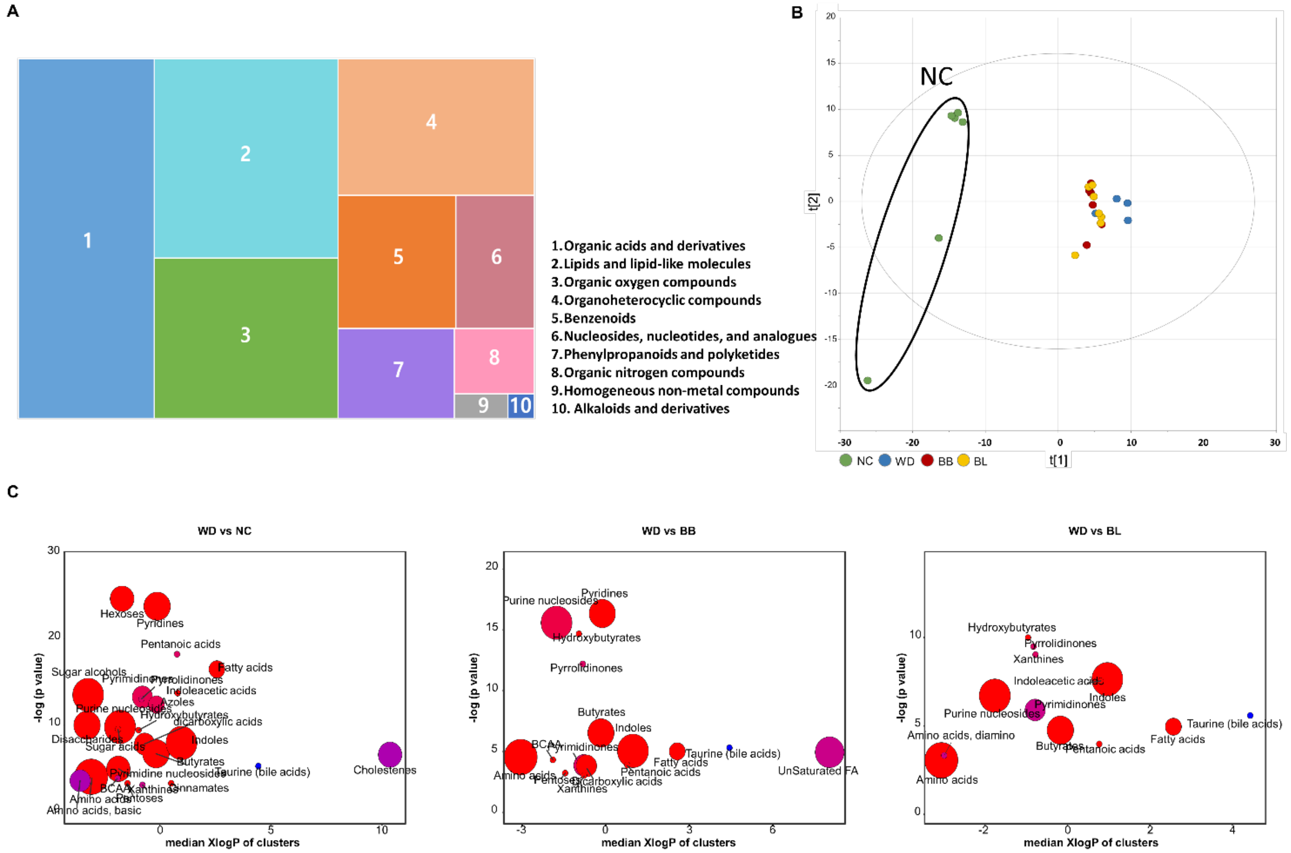

SUPPLEMENTARY FIGURE 2: Mass-spectrometry based untargeted metabolic profiles of mouse cecal samples . (A) Chemical classification of 284 mouse cecal metabolites based on Human Metabolome DataBase (HMDB). The treemap consists of ten rectangles according to the major superclass. The area of the rectangle is proportional to the number of metabolites. (B) The score scatter plot of cecal metabolites based on principal component analysis (PCA). (C) Chemical similarity enrichment analysis of annotated metabolites in NC and probiotics supplement groups compared to WD. The y-axis shows most significantly altered clusters on top, x-axis shows XlogP values of metabolite clusters. NC, Normal chow diet group; WD, Western diet group; BB, WD + *B. breve* group; BL, WD + *B. longum* group.

SUPPLEMENTARY TABLE 1: Summary of univariate statistics on NC vs BB and BL

**Mouse cecal metabolites (indole, tryptophan, SCFAs)**	**p-value (NC VS BB)**	**p-value (NC VS BL)**
4-Hydroxyindole	0.4452	0.2343
Indole	0.5338	0.7308
Indole-3-acetic acid	0.0012*	0.0012*
Indole-3-acrylic acid	0.0513	0.0734
Indole-3-propionic acid	0.0012*	0.0012*
Methyl indole-3-acetate	0.0012*	0.0012*
5-Hydroxyindole-3-acetic acid	0.0012*	0.0012*
Indole-2-carboxylic acid	0.0012*	0.0012*
Indole-3-lactic acid	0.035*	0.8357
Indole-3-pyruvic acid	0.1014	0.035*
Glycodeoxycholic acid	0.0734	0.1375
Lithocholic acid	0.8357	1
Taurocholic acid	0.0513	0.366
Taurodeoxycholic acid	1	0.2343
Cholic acid	0.0012*	0.0012*
Deoxycholic acid	0.0012*	0.035*
Glycocholic acid	0.0012*	0.014*
Taurochenodeoxycholic acid	0.0012*	0.0513
Acetic acid	0.0012*	0.0012*
Propionic acid	0.0012*	0.0012*
Butyric acid	0.0012*	0.0012*
Iso-butyric acid	0.0047*	0.0047*
Valeric acid	0.0734	0.0734
Iso-Valeric acid	0.1807	0.1807

p-value was calculated based on Mann-Whitney U-test. *$p<0.05$
